# Pathways and Barriers for Ion Translocation through the 5-HT_3_A Receptor Channel

**DOI:** 10.1371/journal.pone.0140258

**Published:** 2015-10-14

**Authors:** Danilo Di Maio, Balasubramanian Chandramouli, Giuseppe Brancato

**Affiliations:** Scuola Normale Superiore, Piazza dei Cavalieri 7, I-56126, Pisa, Italy; Dalhousie University, CANADA

## Abstract

Pentameric ligand gated ion channels (pLGICs) are ionotropic receptors that mediate fast intercellular communications at synaptic level and include either cation selective (e.g., nAChR and 5-HT_3_) or anion selective (e.g., GlyR, GABA_A_ and GluCl) membrane channels. Among others, 5-HT_3_ is one of the most studied members, since its first cloning back in 1991, and a large number of studies have successfully pinpointed protein residues critical for its activation and channel gating. In addition, 5-HT_3_ is also the target of a few pharmacological treatments due to the demonstrated benefits of its modulation in clinical trials. Nonetheless, a detailed molecular analysis of important protein features, such as the origin of its ion selectivity and the rather low conductance as compared to other channel homologues, has been unfeasible until the recent crystallization of the mouse 5-HT_3_A receptor. Here, we present extended molecular dynamics simulations and free energy calculations of the whole 5-HT_3_A protein with the aim of better understanding its ion transport properties, such as the pathways for ion permeation into the receptor body and the complex nature of the selectivity filter. Our investigation unravels previously unpredicted structural features of the 5-HT_3_A receptor, such as the existence of alternative intersubunit pathways for ion translocation at the interface between the extracellular and the transmembrane domains, in addition to the one along the channel main axis. Moreover, our study offers a molecular interpretation of the role played by an arginine triplet located in the intracellular domain on determining the characteristic low conductance of the 5-HT_3_A receptor, as evidenced in previous experiments. In view of these results, possible implications on other members of the superfamily are suggested.

## Introduction

**Fig 1:**
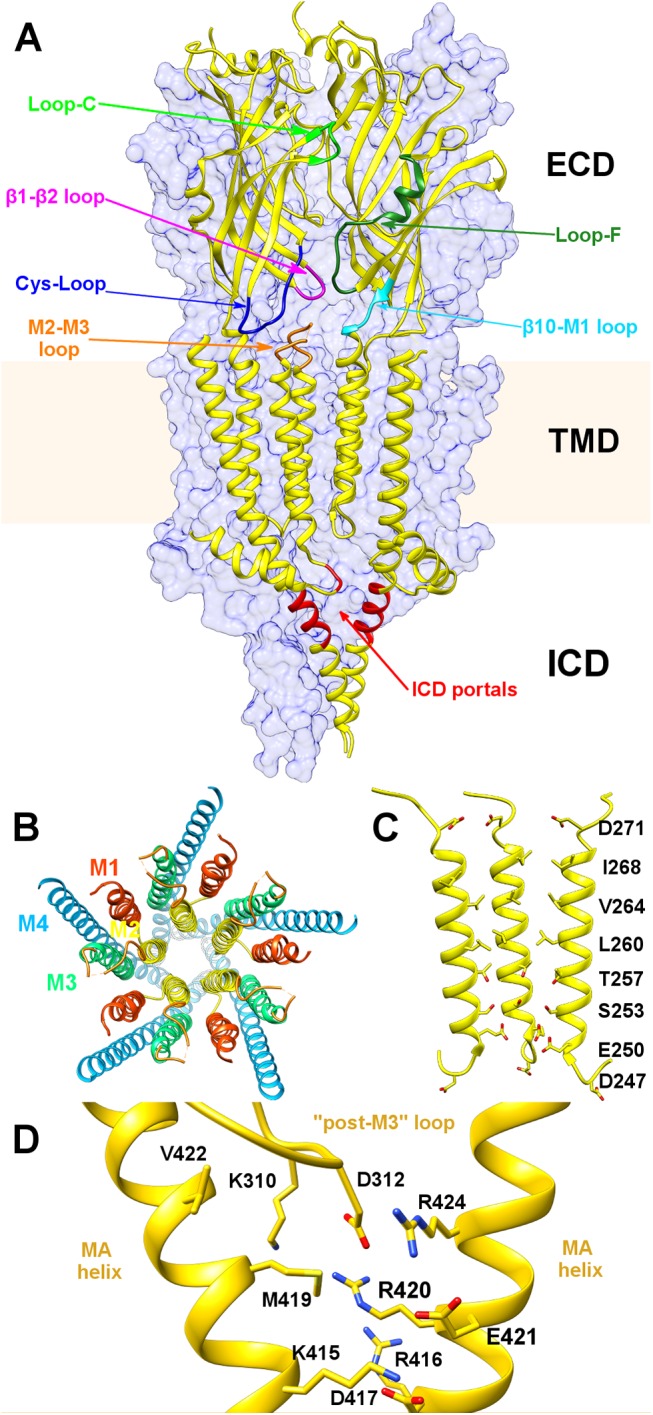
Topology of the murine serotonin 5-HT_3_A receptor (PDB entry: 4PIR). (A) Side view of the homo-pentameric receptor along the channel axis, showing the three main domains (ECD, TMD and ICD). The two front subunits are shown as yellow cartoons. The remaining three subunits are shown as surfaces colored in blue. (B) Top view of the TMD helices shown as ribbons. (C) Side view of the M2 helices and residues that line the channel interior, with side chains displayed as sticks. The two front helices have been removed for clarity. (D) Enlarged view of the residues shaping the ICD portals, shown as sticks.

In recent years, X-ray crystallographic structures of the pentameric ligand-gated ion channels (pLGICs) have boosted the research in computational biology, owing to their importance in vital neuronal activities and their suitability as targets for pharmacological treatments. pLGICs form the so-called Cys-Loop receptor superfamily and are involved in a number of different physiological and pathological roles[1]: in signal transduction processes at synaptic level,[2] pLGICs can change membrane potential by allowing ions to transiently translocate through them via a complex (not fully uncovered) gating mechanism triggered by neurotransmitter binding. Members of this family are integral oligomeric membrane proteins made up by three domains [3,4]: 1) an extracellular domain (ECD), which contains the binding pocket of the neurotransmitter (at the interface of two subunits), 2) a transmembrane domain (TMD) characterized by a bundle of *α-*helices spanning across the membrane and forming the channel core region and 3) an intracellular domain (ICD), which has a role in the localization and regulation of the receptor and whose size could vary among the members of the family (Fig 1A). Since the discovery of the first member of the family, the nicotinic acetylcholine receptor (nAChR), these receptor channels have been intensively studied through a considerable number of experimental [5–8] and, recently, also computational [9–15] approaches. To date the residues forming the binding pocket have been identified,[16,17] as well as those responsible for the channel gating upon agonist binding.[5,7–11,13–15,18–29] Also residues affecting biophysical properties, like ion selectivity and conductance, and pharmacological properties have been discovered.[6,12,30–35] Despite the progress towards the full comprehension of pLGICs, many issues are still unsolved primarily because of scarce information on the conductive channel structures. Besides, it is still unclear the relation between the observed holo-open (conductive) and holo-desensitized (non-conductive) states. As a consequence, most experiments and computational studies rely on the resolved closed-state structures for the interpretation of many pLGICs properties.

Among others, the 5-Hydroxytryptamine receptor channel type 3 (5-HT_3_R), which is widely expressed throughout the brain and in peripheral organs (e.g., the gastrointestinal tract), has been extensively studied from a structural and functional point of view.[36] The 5-HT_3_R is involved in many neurophysiological functions,[37,38] like nervous transmission regulation, pain processing, peristalsis and in some pathological states[38] like chemotherapy-induced nausea and vomiting and various psychiatric disorders. Five different subunits of this receptor have been identified in the genome: among these, only receptors formed by subunits A and B have been extensively investigated and characterized in mammals, while the biological relevance of other forms made up by subunits C, D, and E has not been fully elucidated. From a biophysical point of view, the homo-pentameric 5-HT_3_A receptor is by far the most studied form, due to the capability of subunit A to assemble into functional homomeric channels. Successful experimental efforts on the 5-HT_3_A receptor led to the identification of the residues shaping the binding site,[39–47] the inner surface of the M2 helices[48], which circumscribe the transmembrane portion of the channel, and the membrane associated (MA) helices in the ICD.[49] Moreover, an extensive work has been carried out in order to unravel the nature of the amino acids coupling the agonist binding to the channel gating,[5,8,22,25] as well as those responsible for the cation-selectivity[50,51]. Also, residues responsible for the very low single-channel conductance, which differentiate the 5-HT_3_A receptor from other members of the family, have been identified.[52] However, despite all these efforts, many structural and mechanistic details of 5-HT_3_A still remain poorly understood. In the present work, taking advantage of the recently resolved mouse 5-HT_3_A crystal structure in a putative closed state,[53] we have carried out the first computational study of such a receptor channel to investigate protein dynamics as well as ion-receptor interactions. In particular, through the use of atomistic molecular dynamics simulations and free energy calculations, we have studied three aspects of the receptor: 1) the structural and dynamical features of the whole channel, including ECD, TMD and part of the ICD (MA helices); 2) the potential of mean force of the single-ion translocation through TMD and ICD (the latter formed by post-M3 loops and MA helices) in order to identify and evaluate the energetic barriers experienced by the ions crossing regions pivotal for ionic conductance and selectivity; 3) the molecular determinants of the low single-channel conductance. In the latter case, we have simulated three systems, i.e. a wild-type protein and two high-conductive mutants (namely, the R420D single mutant and the R416Q/R420D/R424A triple mutant, according to the crystal structure numbering adopted in this work). Our overall ~1 μs simulation data provided evidences for the existence of intersubunit lateral pathways at the ECD-TMD interface used by ions to access the channel interior and suggested the possible influence of residues, not investigated so far, on single-channel conductance and cation-selectivity. Moreover, peculiarities, similarities and differences in structural and biophysical properties, as compared to other well-known homologue channels, are thoroughly presented and discussed.

## Results

### Structural Features of the 5-HT_3_A Receptor

In Fig 1A, the structure of the 5-HT_3_A receptor is depicted, where the three domains forming the integral homopentameric membrane protein are evidenced: 1) the ECD is mostly made up by ten β-sheet strands along with the corresponding loops interconnecting them; 2) the TMD is formed by an α-helix bundle of four α-helices per monomer, namely M1, M2, M3 and M4, spanning the lipid bilayer, where the assembly of the internal M2 helices frames the channel core (Fig 1B). The pore walls are characterized, in the central region, by a stretch of hydrophobic residues, while polar and charged residues are located at both ends of the TMD channel (Fig 1C). In particular, it is believed that the gating mechanism, which is triggered by agonist binding, does involve a rather complex series of movements leading to the pore expansion/compression at the TMD level (see, for example, ref.[18] for a recent account of the gating mechanism in pLGICs); 3) the ICD is formed by an α-helix (MA-helix) and a long, partially unresolved, loop connected to the M3 helix in the TMD. The entire 5-HT_3_A receptor was simulated for about 100 ns, without applying an external electric field, after being embedded into a lipid membrane and solvated by adding water and 0.15 M NaCl (details are provided in the Methods section).

First, we evaluated the root mean square deviations (RMSD) of the system with respect to the starting molecular configuration in order to test the system stability throughout the simulated time interval. The RMSD for all the three domains of the receptor, as analyzed separately, was stable (below 1.5 Å) during the entire simulation (S1 Fig), hence the system maintained a stable pentameric assembly over time. Afterwards, we analyzed the main protein structural fluctuations through the evaluation of the root mean square fluctuations (RMSF). The RMSF analysis revealed that the protein most flexible regions are the loops C and F in the ECD and the M2-M3 loop, as shown in S2 Fig, while the rest of the protein appeared, overall, quite rigid.

**Fig 2:**
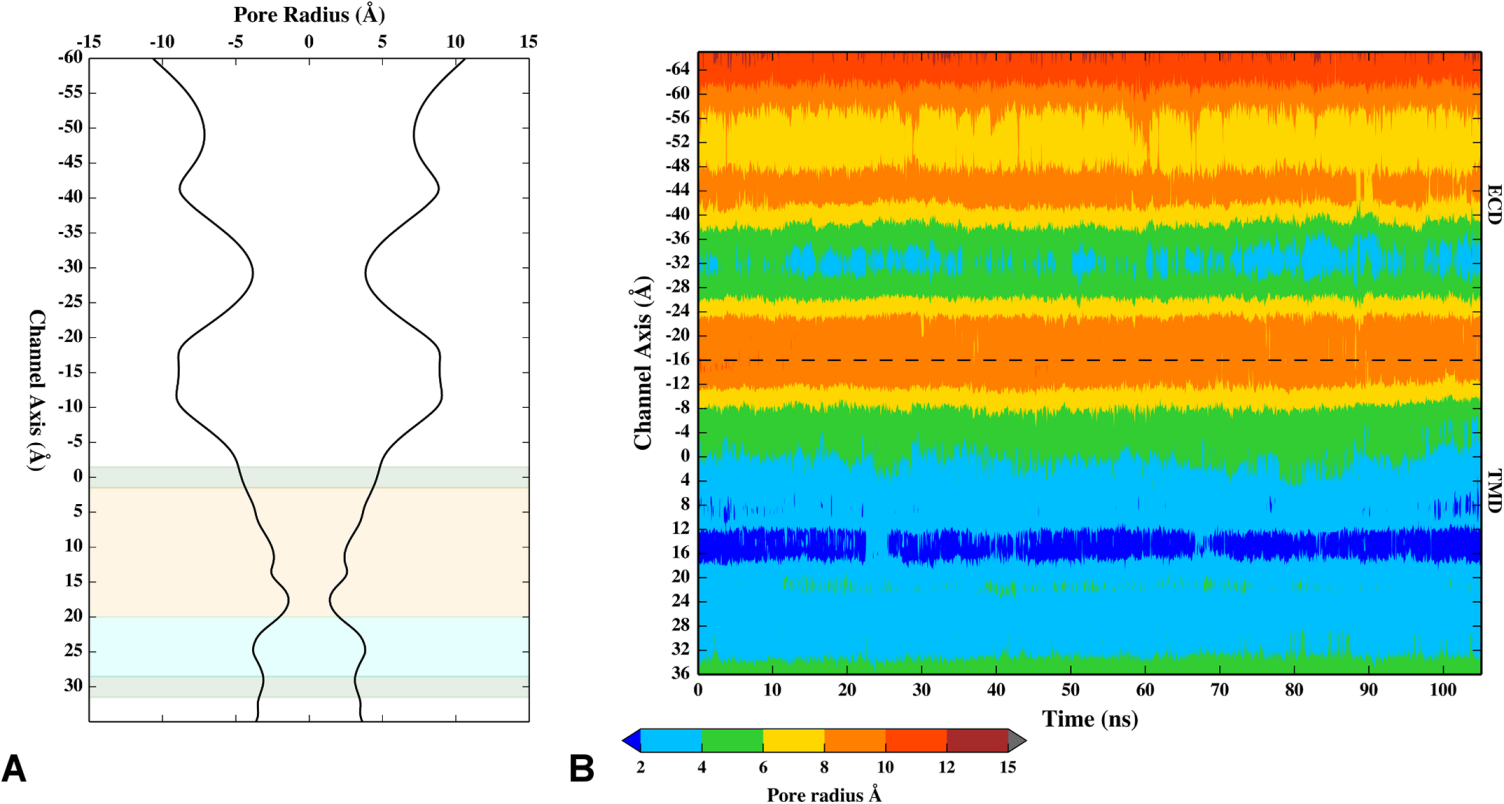
Pore dimension analysis. (A) Average pore radius as a function of channel axis position (mirror symmetry applied for clarity). The shaded bands highlight different regions of the TMD, namely negatively charged (green), hydrophobic (orange) and polar (cyan) residues. (B) Time series of the pore radius as a function of channel axis position. The dotted line identifies the interface between ECD and TMD. In both graphs the zero is taken as the centroid of D271 C_α_ atoms. Negative values represent the extracellular side of the pore, while positive values represent the intracellular side.

We also analyzed the channel dimension along the receptor longitudinal axis (i.e., *z*-axis), in terms of the pore radius as provided by the HOLE program, and found a funnel-like profile with a minimum constriction point located in the TMD (Fig 2A), already pinpointed as the location of the channel gate[9,48]. Note that a first constriction point was observed at position K108 (at *z* = ~ -30 Å) in the ECD. Here, the obtained pore radius is around 3.8 Å, which makes this site almost as constricted as the upper part of the hydrophobic segment in the TMD (at *z* = ~ 5 Å). In the crystal, a sulphate ion from the crystallization liquor was found to interact with the ring of charged K108 residues, hence the K108 side chains adopted an extended conformation towards the center of the channel, considerably shrinking the pore size at this site. In our simulation, the K108 side chains were folded back to interact with the D105 side chain of adjacent subunits (S3 Fig). In the channel hydrophobic region the pore radius at residue V264 and L260 reached its minimum (3.0 Å at V264 and 2.5 Å at L260), making L260 the narrowest point along the TMD. Note that the time evolution of the pore dimension has shown only little deviations from the average structure (Fig 2B) and the initial crystal structure (i.e., pore radius ~3.3 Å at V264 and ~2.3 Å at L260) during the dynamics, supporting an apparent closed state of the 5-HT_3_A channel (i.e., not compatible with the passage of aqua ion clusters). Albeit in the crystal structure the maximum constriction point was reported further down (*z* = ~ 30 Å) at position E250 (i.e., pore radius ~2 Å), the side chains of these residues were not well resolved and were modeled as most plausible rotamers. Therefore, the reported pore size estimate has to be considered rather uncertain. From our MD simulation, we have obtained, on average, a pore radius of about 3.3 Å at residue E250, which is perhaps a more appropriate estimate considering the effect of side chain repulsion.

### Ion Permeation into the Receptor Body

**Fig 3:**
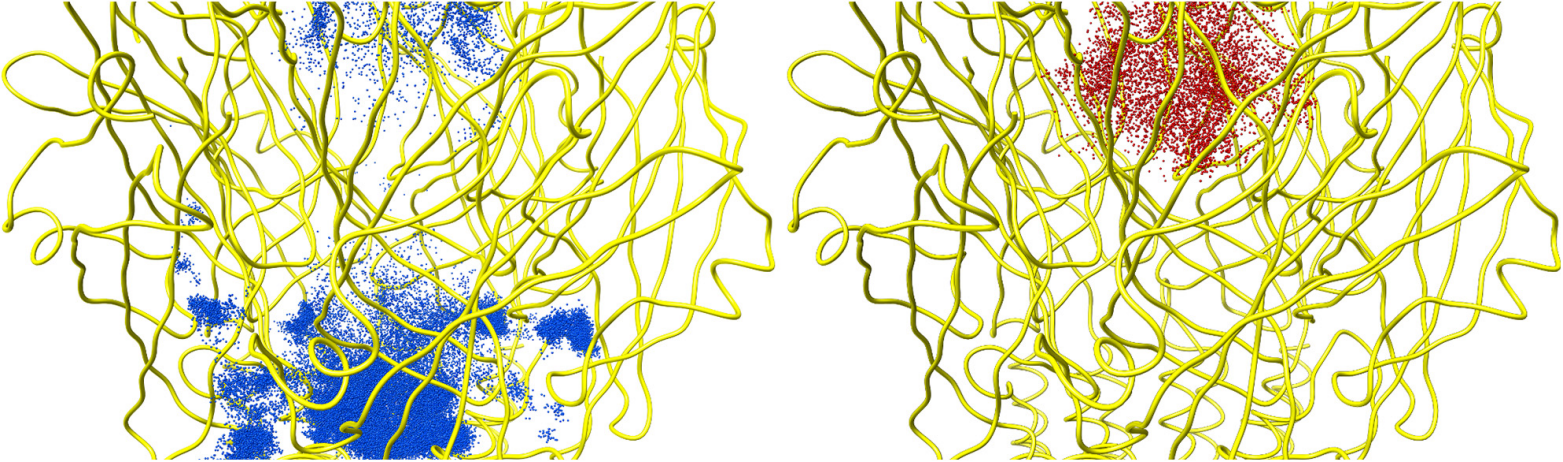
Ion distribution inside the 5-HT_3_A channel. Na^+^ (blue) and Cl^-^ (red) ion positions, as issuing from the MD the simulation, sampled inside and outside the receptor (a cylinder of 25 Å radius has been defined, taking the centroid of D271 C_α_ atoms as the center). Note, at the bottom of left panel, the distribution of Na^+^ ions permeated into the receptor through the ECD lateral pathways. The average structure of the protein is shown as yellow ribbons.

**Fig 4:**
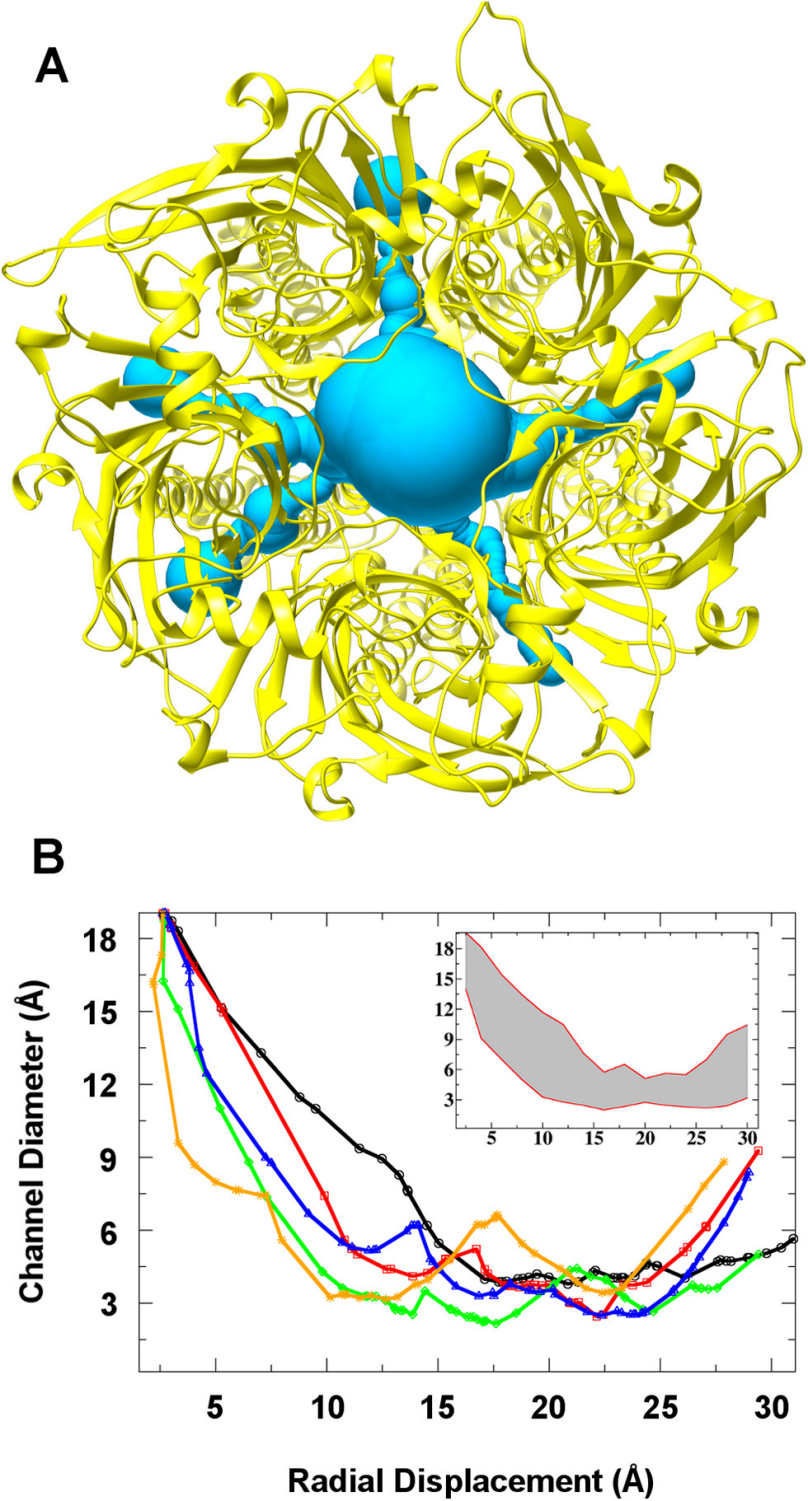
Intersubunit lateral ion pathways. (A) Top view of the 5-HT_3_A receptor principal and lateral channels predicted by MolAxis, shown as a series of blue spheres with diameter proportional to the accessible space, and (B) the corresponding lateral pore dimensions (the inset displays the maximum channel diameter deviations from the average (grey band) as issuing from the analysis of several protein structures). The protein average structure is depicted in yellow cartoons.

The ion permeation through the 5-HT_3_A receptor was evaluated by considering an initial configuration with no ions within the entire protein body and then by monitoring the ion entrance through the accessible pathways. A cylindrical region of 25 Å radius aligned along the channel axis, including the whole ECD, TMD and part of the ICD, was considered in order to follow the permeation events. Note that ion permeation was observed only from the extracellular side of the receptor. Fig 3 depicts the distribution of ion (Na^+^ and Cl^-^) positions sampled during the MD simulation. It is apparent that the presence of Cl^-^ inside the ECD vestibule of the channel is essentially smaller than that of Na^+^, though not negligible. Interestingly, we have observed that the top side of the channel is not the only way through which ions may access the channel ECD vestibule. Indeed, cations can also enter into the ECD passing through five lateral channels located at the interface between the ECD and TMD of any pair of adjacent subunits (as shown in the S1 Video). Such channels are shaped by residues belonging to Cys-Loop, β1-β2 loop, M2-M3 loop, β10-M1 loop and the C-terminal part of the F-loop: to be specific, the lateral channels are formed by residue D52, E53, K54 and N55 from the β1-β2 loop, by residue Q184, G185, E186 and, to a minor extent, E188 from the F-loop C-terminal part, by residue R219, P220, L221, F222 and, to a minor extent, R217 and R218 from the β10-M1 loop, by residue C135, S136, L137, D138 from the Cys-Loop and a stretch of residues (i.e., P274-T280) from the M2-M3 loop (S4 Fig). From our MD simulation, such lateral pathways appeared to be the main entering route to the ECD vestibule: we counted at least 5 different permeating Na^+^ ions passing through the lateral channels against one Na^+^ ion entering from the channel top side (note that we have observed Na^+^ ions passing through at least three of the five available lateral channels, which are all equivalent due to symmetry). The presence of only one top entryway versus five equivalent lateral ones, though featuring a smaller diameter, may suggest a detectable contribution of the latter to the overall single channel conductance. In order to better characterize the size of such lateral cavities, we submitted an average receptor configuration issuing from the MD simulation to the MolAxis webserver. Fig 4 displays the lateral channels and their corresponding diameter size, as provided by the MolAxis analysis tool. Since the maximum constriction point lies below the diameter of Na^+^ first solvation shell (about 6 Å), we concluded that these channels are not constitutively open, but may transiently get expanded to allow ion passage (see inset of Fig 4). Motivated by the present results, we wondered if such structural features could be common to other 5-HT_3_A receptor analogues. Similarly, we analyzed the known structure of nAchR (PDB entry: 2BG9)[54], GluCl (PDB entry: 4TNV)[55], ELIC (PDB entry: 3RQU)[56], GLIC (PDB entry: 3P50)[57] and finally 5-HT_3_A crystal structure (PDB entry: 4PIR) as a reference, after modeling the missing part of the M2-M3 loop. Surprisingly, MolAxis predicted the existence of such channels not only for the 5-HT_3_A crystal structure but also for nAchR and GluCl, whereas they have not been found in GLIC and ELIC (S5 Fig).

Then, we analyzed the ion distribution along the 5-HT_3_A channel axis in terms of the average occurrence number (S6 Fig). Results have shown an extended region roughly spanning 35 Å along the pore axis, where neither chloride nor sodium ions were found; this region corresponds to the TMD central region, comprising both a purely hydrophobic (from I268 to L260 of M2 helix) and a polar (from T257 to E250 of M2 helix) stretch, and the first part of the ICD. At the beginning of the TMD (around *z* = 0 Å), it has been observed a spike in the average number of Na^+^, while the presence of Cl^-^ was basically null. Here, just before the hydrophobic region characterizing the TMD pore, a ring of aspartates (D271) wards a funnel-shaped zone which seems well suited to act as a pool for concentrating cations before their subsequent translocation along the channel, as already proposed in previous studies on cation selective channels.[12,58,59] In light of these results, we decided to investigate in more detail the role played by the TMD in hindering the ion passage in the closed state and in selectively filtering cations versus anions.

### Free Energy Analysis of Ion Translocation through the TMD

**Fig 5:**
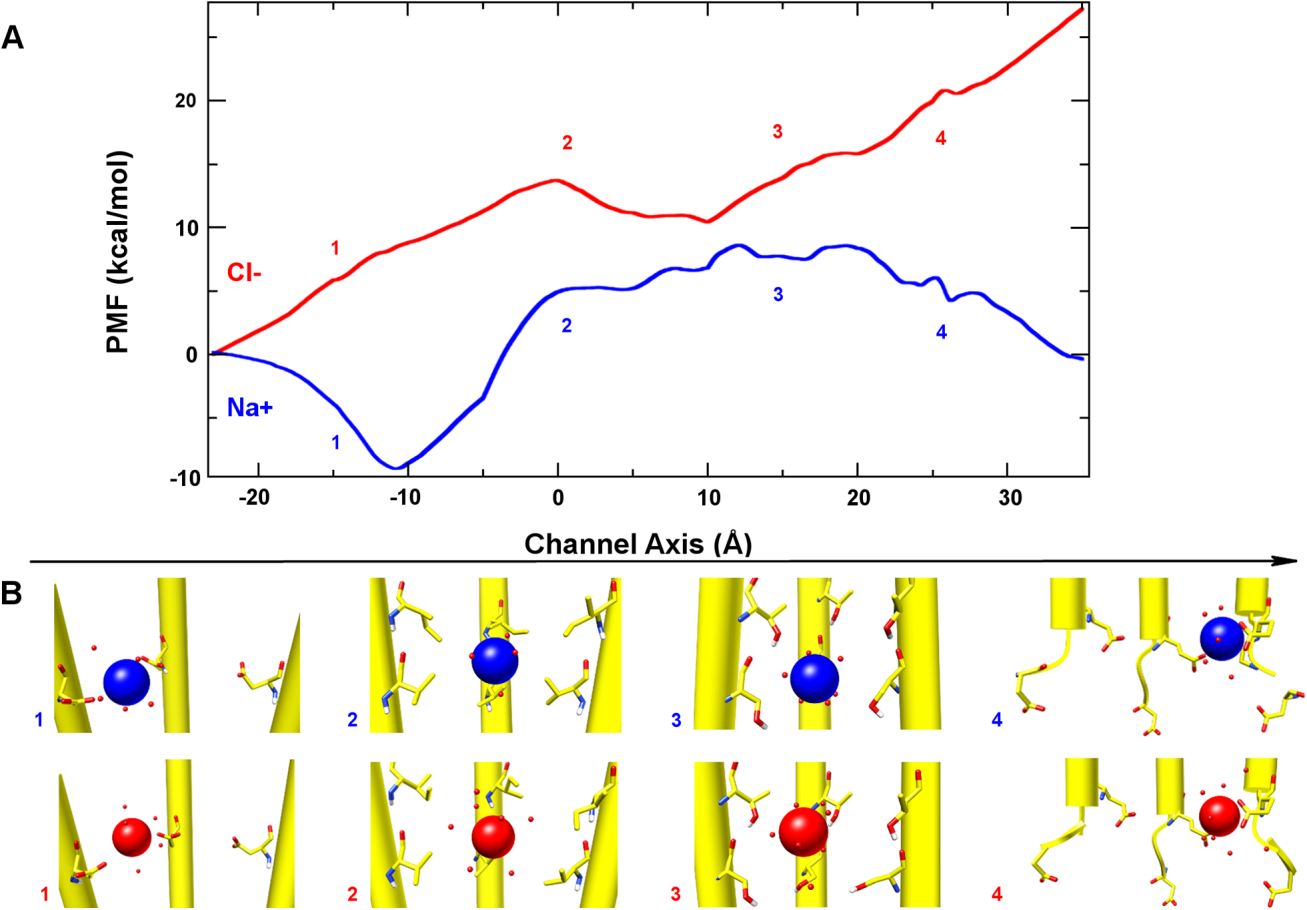
Free energy barriers to ion translocation. (A) Potential of mean force profiles for Na^+^ and Cl^-^ along the channel axis. Channel axis origin is set to the centroid of V264 C_α_ atoms, at the center of TMD. Negative values refer to the extracellular side, while positive values refer to the intracellular side. The numbers (1–4) in the plot indicate: (1) negative, (2) hydrophobic, (3) polar and (4) negative regions of the pore, as described in the text. (B) Representative snapshots of Na^+^ (blue) and Cl^-^ (red) along the TMD pore.

In order to investigate the role of TMD residues on ion transport properties, we evaluated the single-ion potential of mean force of both Na^+^ and Cl^-^ inside the TMD pore. The present analysis is limited by the availability of only the closed state 5-HT_3_A structure. However, as shown in several previous studies on pLGICs closed-state channels,[9,12,58] a general qualitative picture of the pore can be satisfactorily obtained, including some important features such as a molecular description of the selectivity filter. Furthermore, we complemented such an energy analysis with a detailed picture of the variable ion coordination number along the pore, evaluating the average number of water molecules and side-chain atoms in the first solvation shell (S7 Fig). To this end, a subsystem of the whole receptor protein made up by only the TMD and ICD was obtained from the mouse 5-HT_3_A crystal structure and embedded into lipid membrane and water. Apart from a few ions ensuring the system electric neutrality, only one ion (either Na^+^ or Cl^-^) was inserted in the TMD for carrying out the free energy analysis and the resulting system simulated for an overall time of about 240 ns (see Methods for details). Fig 5 shows the obtained PMF profiles for both considered ions. At the extracellular end of the TMD, both Na^+^ and Cl^-^ experience a set of negatively charged residues, namely D271 at position *z* ~ -10 Å along the channel axis. The Na^+^ PMF shows a pronounced minimum in this region (~ -9 kcal), which is also characterized by a relatively wide pore diameter (~9.5 Å). This result supports the hypothesis that this zone may serve as a funnel for cations,[12] increasing their local concentration before permeating into the narrowest portion of the channel. Conversely, the marked increase of the Cl^-^ PMF (~ 9 kcal) in the same region indicates that the negative residues effectively do repel anions from the pore and thus disfavor their further translocation through the channel. Below residue D271, the M2 helices expose hydrophobic side chains (I268, V264, L260) towards the interior of the pore. The PMF in this region (from *z* = -5 to +10 Å along the channel axis) reaches a local maximum for both ions in correspondence of V264 (~ 5 kcal and ~14 kcal for Na^+^ and Cl^-^, respectively, around position *z* = ~ 0 Å along the channel axis). At this channel location, both Na^+^ and Cl^-^ lose, on average, around one water molecule from their first solvation shell without compensating this loss with side chains interactions. These results corroborate the hypothesis regarding the hydrophobic nature of the gate, previously proposed on the basis of experimental and computational studies on homologue receptors,[9,60] and are in qualitative agreement with previous PMF calculations performed on the α7 acetylcholine receptor.[12] In the polar region of the TMD, lined between residue T257 and S253, (from *z* = +10 to +20 Å along the channel axis) the PMF profile is little affected in the case of Na^+^, while it keeps increasing for Cl^-^. A careful inspection at the ion coordination profile (S7 Fig) shows that in the case of Na^+^ the average number of coordinating species remained about 5 (i.e., the loss of water molecules is compensated by the close interaction with T257 and S253 side chains), thus supporting the rather flat PMF observed in this region. Conversely, Cl^-^ has lost more water molecules than acquired polar side chains in its first coordination shell and, in turn, this led to a PMF increase while going towards the ICD. Note that the free energy of ion translocation in the present narrow pore segment could be also related to the difference in first solvation shell size between Na^+^ and Cl^-^. To proceed further, after residue S253, the pore presents two rings of negatively charged residues, namely E250 and D247, located at the end of the M2 helix and on the M1-M2 loop, respectively (from z = +20 to +35 Å along the channel axis). In previous studies on the *Torpedo* nAchR and other homologue protein models built up from this structure,[9,12] the interpretation of the computed single-ion PMF at the intracellular end of the TMD turned out to be problematic, owing to the poor structural resolution of the nAchR channel at this site and to the lack of the M3-M4 loop, which does interact with the M1-M2 loop affecting the conformation of the latter. On the other hand, the 5-HT_3_A crystal structure presents both a higher atomic resolution and more complete portion of the M3-M4 loop. In turn, this allowed us to obtain a more accurate estimate of the PMF, as compared to previous studies. In this region of the pore, Na^+^ and Cl^-^ PMF profiles reflected opposite electrostatic interactions with E250 and D247. On one hand, these interactions further destabilize the passage of Cl^-^ ions inside the pore, and on the other hand they stabilize significantly Na^+^ ions. Accordingly, at this site the two PMFs show the maximal deviation: not surprisingly, residue E250 has been identified as the main selectivity filter of the 5-HT_3_A channel based on conductive state experiments. Our findings also suggest an important role for D247, especially for providing the exit driving force for Na^+^ ions from the TMD, and further considerations are provided in the Discussion.

### Mutation Effects on the ICD Portals

**Fig 6:**
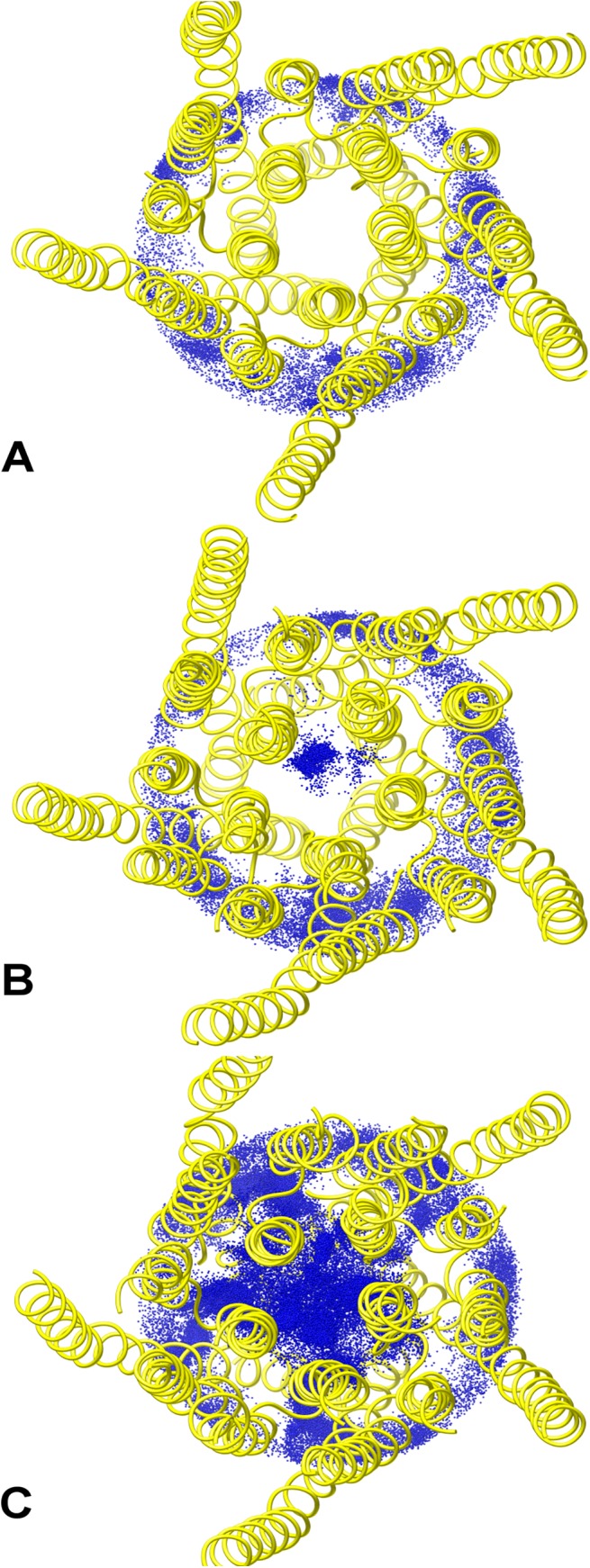
Ion distribution at the ICD level. Distribution of Na^+^ ion positions (blue dots) extracted from the (A) WT, (B) R420D and (C) QDA simulations inside and outside the ICD vestibule (within a cylinder of 20 Å radius, taking the centroid of V264 C_α_ atoms as the center). The protein average structures are depicted as yellow ribbons.

**Fig 7:**
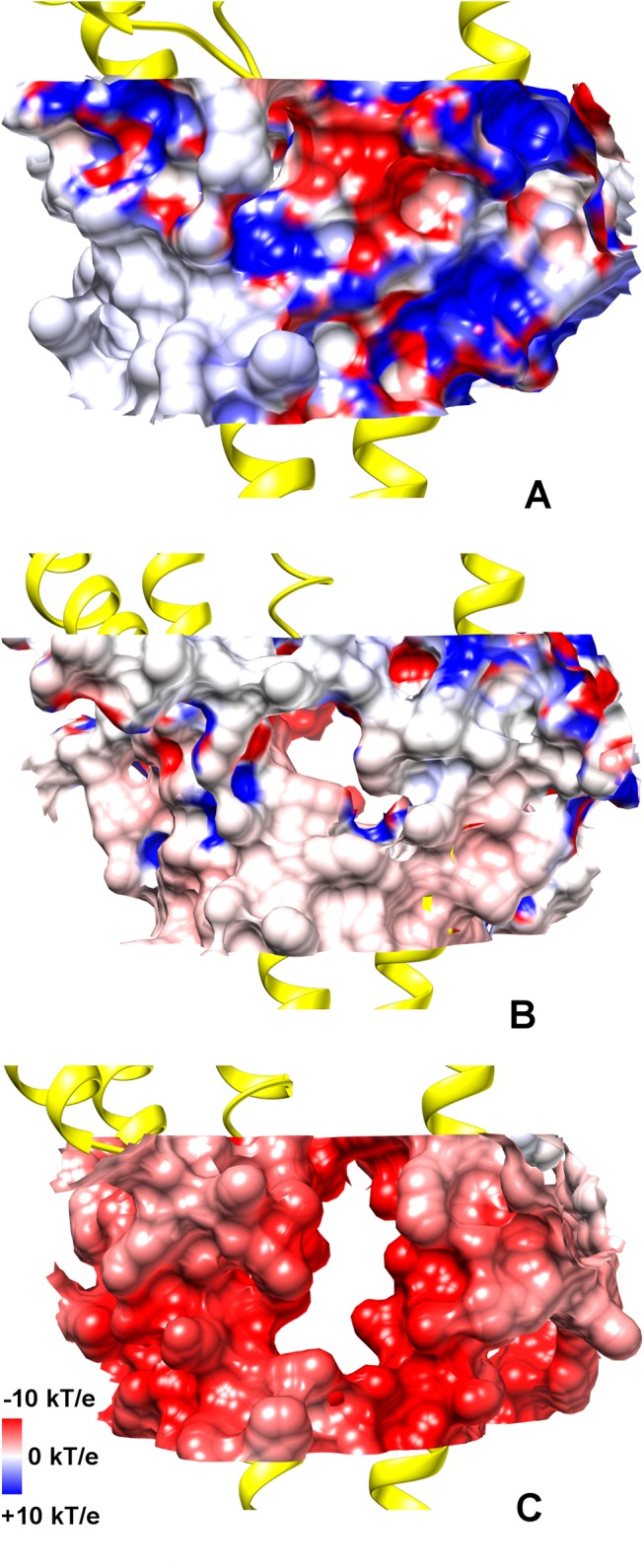
ICD portals. Electrostatic and steric properties of the ICD portals as issuing from (A) WT, (B) R420D and (C) QDA systems. The ICD portals are depicted as molecular surfaces, colored according to their electrostatic potential from blue (positive) to red (negative). The average structures of M3 helix, post-M3 loop, MX and MA helix, from one subunit, and MA helix from an adjacent subunit are shown in yellow cartoons.

In a seminal study on the human 5-HT_3_A receptor,[52,61] whose results have been later confirmed on the mouse 5-HT_3_A receptor,[62,63] it has been shown that the replacement of an arginine triplet (R416, R420, R424) located in the MA helix with corresponding residues from the 5-HT_3_B subunit markedly affected the single-channel conductance. This evidence suggested that such mutations could facilitate the ion passage through lateral portals formed by adjacent MA helices,[61] otherwise hindered in the wild-type protein. These lateral openings are molded by a small stretch of residues belonging to the MA helices, i.e. 415–424, as identified by previous functional studies.[49] In particular, the portals are lined by residue K415, R416, M419 and V422 from one subunit, and residue D417, E421, R420 and R424 from the adjacent one (Fig 1D). The “ceiling” of the portals is formed by the starting portion of the M3-M4 segment (henceforth referred to as the “post-M3” loop, following the nomenclature reported in ref.[53]), especially by residue K310 and D312. Hydrogen-bond and salt-bridge networks among such residues apparently make the ICD cavities closed in the native receptor. In this study, we investigated in some detail the ICD structure of the mouse 5-HT_3_A channel, which is characterized by an unusual large fraction of well-resolved atomic coordinates, in order to better describe the proposed hampering effect towards ion permeation modulated by the present arginine triplet. Inspired by the findings of the mutagenesis experiment mentioned above (and in the absence of known structures of the mutants probed in that study), three model 5-HT_3_A receptor systems made up solely by TMD and ICD were created, namely one wild-type (WT), one single mutant (R420D) and one triple mutant (R416Q/R420D/R424A, hereafter referred to as QDA). All systems were simulated for about 100 ns under physico-chemical conditions mimicking the natural environment (see Methods for details). Although part of the M3-M4 loop is truncated in the 5-HT_3_A crystal structure, the present ICD structure represents the most complete model for studying ion diffusion through intracellular portals. Fig 6 shows the distributions of Na^+^ ion positions, sampled during the simulations, inside and outside the ICD for the three considered systems. In WT, no ions have been observed to pass from the aqueous solution to the intracellular vestibule; in the R420D mutant, one ion was able to translocate through the portals in the considered time interval and, in QDA, ions freely diffused from the solution into the ICD. To test if such differences in ion diffusivity were the result of structural changes or differences in the domain dynamics upon mutation, we calculated the RMSD and the per-residue RMSF on backbone atoms, especially focusing on those residues lining the portals walls, as described above. The RMSD for the three systems was essentially indistinguishable, with differences of less than 0.4 Å and overall similar broadness (S8 Fig). Similarly, the RMSF has not evidenced significant differences in structural fluctuations (S9 Fig). Taken together, these results rule against the hypothesis that arginine replacement may impinge on the MA helix dynamics, suggesting, on the contrary, that more localized effects do account for the observed ion permeation. Consequently, we have examined if changes in local electrostatics and/or side-chain structural rearrangements could be the determinants for portal opening. Fig 7 shows how the sequential mutation of arginines with smaller residues, either negatively charged or neutral, has induced an increasing portal expansion along with a distinct change in the electrostatic potential of the cavities from mostly positive to mostly negative. In WT, residue D312 from the post-M3 loop may strongly interact with two arginines of the MA helix (S10 Fig), thus anchoring the positively charged side chains of the latter and effectively occluding the lateral portals due to steric hindrance (Fig 7A). On the other hand, in R420D mutant, the observed partial expansion (Fig 7B) is sufficient to allow ion permeation, though close interactions of residue D312 and D420 with the two remaining arginines (i.e., R416 and R424) are apparent. In QDA, no more stable electrostatic interactions involving residue D312 are possible and, as a consequence, the mutated residues do not concur to obstruct the ICD portals (Fig 7C). In particular, going from WT to R420D and QDA, we have observed a gradual increase in the D312 side-chain flexibility (S10 Fig), which, in turn, reflects the opening of the portals for the reasons sketched above.

## Discussion

Atomistic MD simulations of the 5-HT_3_A receptor have provided several insights into its structural features and interaction with permeating ions, suggesting interesting connections to channel properties. Although the investigation of a closed-state structure prevents the full comprehension of the role played by some key residues in the conductive conformation, our study, similarly to many other studies on pLGICs, contributed to gain further valuable information on the properties of this channel. Besides, as discussed in the following, results are generally in good agreement with recent experimental findings on the same 5-HT_3_A channel and with previous computational studies on homologue proteins.

Our investigation identified a few relatively flexible regions of the protein. In particular, the observed high mobility of loop C is considered crucial for receptor function. It is believed that the outward and inward movement of loop C is pivotal to accommodate the neurotransmitter and to activate the receptor,[64] thus characterizing the protein functional state. Indeed, our simulation showed that in one subunit loop C adopted, for most of the time, an open-lid conformation, whereas several partial opening and closing events occurred in loop C of other subunits, in agreement with previous studies.[11,13,59,64–68] Loop F was also found to be flexible as compared to the rest of the protein, especially in the region spanning residues S176 to E186. This loop has been proposed to act as a flexible structure, adopting different conformations suitable for the interactions with ligands of different size.[46] In many crystallization studies of AchBP, this loop was only poorly resolved owing to its disordered structure. Another observed flexible part of the receptor is the M2-M3 loop, which in fact is poorly resolved in the 5-HT_3_A crystal structure. It is worth noting that a number of studies indicated that this loop, along with the interacting region between ECD and TMD, is involved in the complex cascade of movements leading to channel gating.[5,7,19,22,26]

Remarkably, we found that the ECD-TMD interface shapes five inter-subunit lateral cavities, as formed by Cys-Loop, β1- β2 loop, loop F C-terminal part, β10-M1 loop and M2-M3 loop, through which ions can reach the interior of the channel from the extracellular medium. This observation prompted us to re-examine other 5-HT_3_A receptor homologue structures. Surprisingly, we detected similar fenestrations also in other members of the superfamily: in particular, these structural features are shared by all tested eukaryotic receptors. Since loop F shows the lowest degree of conservation amongst the six binding site loops, it is tempting to speculate that it could play a role in regulating the cavity size in different receptors, depending on its specific residue sequence. In GluCl, the C-terminal part of this loop is folded to form an α-helix turn, splitting the lateral channel into two channels, both narrower than that of nAchR and 5-HT_3_A. However, a molecular comprehension on the differences among different receptors and the possible effect of mutations on such lateral pathways demands further investigations.

Overall, the pore dimension along the channel longitudinal axis, as issuing from our MD simulation, was in good agreement with the one estimated from the 5-HT_3_A crystal structure:[53] the only two significant differences (K108, E250) have been ascribed to biased side chain conformations of the latter structure. In our simulation, we observed a pore diameter of ~7.5 Å at the most constricted point of the ECD, corresponding to residue K108 (S3 Fig). Note that this evidence is consistent with the observed smaller permeability of the ECD top side with respect to the lateral channels at the ECD-TMD interface, though the absence of electric field, the relatively limited sampling and the use of a closed structure do not allow to draw a clear distinction between such entrance pathways. Besides, it is known that negatively charged MTS reagents can pass through this region and reach the TMD.[48,69] In any case, our results suggest that residue K108, if mutated, might have a detectable influence on ion translocation, perhaps more than D105, which was previously shown to affect the channel conductance.[70] As expected, the maximum constriction point of the channel was found in the TMD hydrophobic region. Here, the channel size was not shown to change significantly during the simulation, thus forming a pore unsuitable to the passage of solvated ions, in line with the hydrophobic gating hypothesis proposed in homologue proteins.[67]

Moreover, we investigated the complex nature of the cation-selectivity in this receptor. Several studies suggested that the selectivity filter is not located in the ECD, though mutations in this domain were proved to affect single-channel conductance.[6,70] On the other hand, there is a general consensus about the notion that the filter is located at TMD level.[51,71] Our free energy calculations on ion translocation suggested that the filter could be “delocalized” between the extracellular (D271) and the intracellular (E250, D247) side of the TMD. However, in α7 nAchR, only homologue residues to E250 and D247 were shown to be important for ion selectivity[30,31] and, in 5-HT_3_A, the contribution of E250 was shown to be dominant.[51,72] Such an apparent discrepancy between theory and experiments can be reconciled by noting that the PMF analysis was performed on a closed-state channel. Moreover, the most agreed hypothesis on the gating mechanism predicts a peculiar rearrangement of the *α-*helices shaping the channel. On this regard, according to the known GLIC and GluCl open-state channel structures[57,73] and to the gating model recently proposed in ref.[10], the gating mechanism does involve the tilting of the M2 helices with respect to the channel axis. In particular, channel opening is characterized by an outward polar tilting of the M2 helix upper segment (above residue 264), with the lower segment mostly unaffected. These conformational changes lead to an increase of D271 inter-residue distances, while those among E250 residues remain substantially unaltered. In this scenario, the funnel located at the extracellular side of the TMD gets broadened, thus weakening the possible electrostatic interactions between D271 and the translocating ions. Moreover, the funnel widening allows, in principle, more Na^+^ ions to be accommodated, with the result of a more effective screening of D271 residues negative charges. Altogether these two effects may account for a minor contribution of D271 to ion selectivity. On the contrary, the lower portion of the M2 helix (below residue 264) does form a narrow pore, which is essentially similar in both closed and open states. Hence, in the open state residue E250 becomes the paramount determinant for ion selectivity, as overall suggested by experiments on this protein and homologue receptors. It should be noted, however, that the role of this residue on the ion selectivity of all Cys-Loop receptors is still a matter of debate due to contrasting mutagenesis experiments on GABA_A_ and GlyR.[32,33]

The PMF analysis suggests another interesting feature of this channel. Our PMF profile supported a considerable contribution of residue D247 to ion translocation, along with the nearby residue E250. Therefore, it would be interesting to test if D247 could affect single-channel conductance as well as residue E250, or even ion selectivity, especially in the case of the 5-HT_3_AB heteromeric receptor, which is characterized by fewer negative residues at position 250 than the corresponding homomeric receptor. Note that the corresponding residue in nAchR was shown to be important for single-channel conductance[6] (however, since in human 5-HT_3_A D247 is replaced by an asparagine, these contributions may not be shared by all organisms). Moreover, the Na^+^ ion attraction by residue D247, while passing through the narrow hydrophobic channel towards the intracellular vestibule, provided an additional driving force to exit the hydrophobic region and the E250 ring. In turn, this may suggest that a multi-ion transport mechanism is not required for ion translocation through the 5-HT_3_ channel, as observed in other channels.[74] Nonetheless, a rather contrasting scenario is expected with divalent cations, considering that position 250 was shown to be a divalent ion binding site with a resulting decrease of ionic conductance in both nAchR and 5-HT_3_.[72,75] Concerning the obtained trend of the single-ion PMF within the TMD pore, we observe that our results are qualitatively in good agreement with previous calculations on homologue channels[12] (taking into considerations possible differences in the simulation protocols), and possibly more accurate in describing the ending region of the pore owing to a better description of the initial ICD region, as pointed out in the Results section.

Finally, we studied the effect of replacing the MA helix arginine triplet, which is typical of the 5-HT_3_A subunit, on cation permeation. Our simulations showed that an increased ionic passage through the ICD portals is achieved by progressively replacing the arginine residues with corresponding residues from the 5-HT_3_B subunit. Such a trend nicely correlates, at least qualitatively, with the enhanced single-channel conductance observed while going from the wild-type to the R420D and QDA mutants and further supports the special role of the arginine triplet in determining the characteristic low-conductivity in 5-HT_3_A channels, as compared to other homologues.[52] Moreover, our study set out to demonstrate that the hampering effect on ion translocation by the MA helix arginines is due to a combination of electrostatic and steric effects, via the formation of stable side-chain interactions, a result well consistent with an hypothesis based upon cystein substitution and subsequent modification by MTS reagents.[76] Supported by our findings, we propose that D312 in the post-M3 loop, so far never investigated, plays an important role in stabilizing the arginine triplet in a conformation preventing the cavity opening. However, the present observation is at variance with a recent hypothesis, based on constrained geometric simulations and mutations of MA negatively charged residues, that considers the increase in MA helix flexibility, not observed in our study, as the main cause of the increase of ion conductance.[77] Please note that the increase in conductance reported in the study mentioned above does not necessarily rule out the importance of the electrostatics of the portals. Indeed, two of the three mutated residues, i.e. E434 and D441, being located on different position on the MA helix, actually point towards the exterior of the protein and are solvent exposed. Thereby, their contribution to the conductance could be somehow indirect.

Interestingly, no ion passed through the ICD portals in the WT system within the simulated time interval, though the trypsin-treated 5-HT_3_A receptor generated to obtain the crystal structure displayed a higher conductance than the unproteolized receptor. This intriguingly suggests also the possible transmission of (a certain degree of) structural changes from the TMD to the ICD upon gating, as previously conjectured.[78] While a quantitative relationship between the experimental conductance of the trypsin-treated receptor and the extent of portal opening observed in our simulations could not be established, we can reasonably assume that the ICD portal structure, especially the *α-*helices, undergoes only a slight rearrangement upon gating, as previous experimental studies seem to suggest.[49,61,76,77] If this is the case, the structural differences among mutants observed in this study could be considered relevant also in the channel conductive state.

## Methods

### 5-HT_3_A Receptor Models

#### Model of the whole 5-HT_3_A receptor

The 5-HT_3_A receptor (PDB entry: 4PIR) was first oriented and then inserted into a POPC (1-Palmitoyl-2-Oleoyl-sn-Glycero-3-Phosphatidylcholine) lipid bilayer using the OPM[79] and CHAMM-GUI[80] servers, respectively. The protein-lipid assembly was then solvated with TIP3P water molecules and Na^+^ and Cl^−^ ions to neutralize the overall charge and obtain a final ion concentration of about 0.15 M, using the VMD[81] software. Ions were introduced at least 8 Å away from the protein. Since a long part of the M3-M4 loop was absent from the structure, the C-terminus of the post-M3 segment and the N-terminus of the MA helix were capped with a methylamine and an acetyl groups, respectively, so as to mimic a more realistic situation where these segment termini are neutral. The M2-M3 loop segment that was unresolved in the crystal structure has been reconstructed using the Modeller program (v9.12).[82] All histidines were set in neutral form, since none of them was found in a context such to justify a protonated state. The resulting simulation box size was 154 Å × 154 Å × 214 Å and the total system size about 400,000 atoms.

#### Equilibration

The complete 5-HT_3_A receptor model, once solvated and embedded into the lipid membrane, was minimized in two steps: first, a 20000 step minimization run was carried out with restraints on all protein atoms (5 kcal/mol/Å^2^); then, a further 15000 step minimization was carried out with 1 kcal/mol/Å^2^ restraints on C_α_ atoms only. Afterwards, the system was slowly heated up from 1 to 310 K over a 4 ns MD simulation, applying gradually decreasing restraints on the protein C_α_ atoms, from 5 to 1 kcal/mol/Å^2^. After such a thermalization, the system was equilibrated for further 2 ns, gradually reducing to zero the atomic restraints on the protein flexible parts (ECD, loops, ICD).

#### Wild-type and mutants of the 5-HT_3_A channel (TMD+ICD)

The procedure to prepare the smaller systems made up by TMD and ICD was slightly different. We first created the mutants starting from the full 5-HT_3_A crystal structure (PDB entry: 4PIR). The three systems (WT, R420D and QDA) were then embedded into the lipid membrane, solvated, thermalized and equilibrated as described above. After equilibration, we created a reduced system (starting at P213, in the β10-M1 loop), removing the ECD from protein structure, capped P213 N-terminus and adjusted the box volume in order to ensure, at least, a 20 Å thick water layer. Ionic concentration was set again to 0.15 M. Special attention was paid to avoid ion inclusions inside the ICD vestibule. The resulting simulation cell was 156 Å x 154 Å x 148 Å, with about 260,000 atoms. Moreover, the three systems were further equilibrated for 2 ns prior to production runs.

### Molecular Dynamics Simulations

#### Simulation details

All MD simulations were performed using the NAMD v2.9 program.[83] Protein, water and ions were modeled with the CHARMM27 force field[84] with CMAP corrections for backbone atoms and NBFIX correction for Na^+^ ions,[85,86] while the CHARMM36 force field[87] was used for lipids. On the C_α_ atoms of only TMD helices, soft symmetry restraints available in NAMD, with a force constant of 0.75 kcal/mol/Å^2^, were applied. ECD, ICD and other more flexible parts of the protein (e.g. loops) were kept unrestrained. It is worth noting that these restraints do not cancel out global movements of the TMD, but rather ensure, on average, a symmetrical homopentameric assembly, as expected in the present system. Simulations were performed under constant pressure (1 atm) and temperature (310 K), where the NPT ensemble was enforced by the Langevin piston pressure control and the Langevin damping dynamics using periodic boundary conditions. All covalent bonds with hydrogen atoms were kept rigid using the SHAKE algorithm. The bonded interactions and the short-range non-bonded interactions were calculated at each time-step (2 fs), whereas the particle mesh Ewald method was used to update the long-range electrostatic interactions every two time-steps. PMEGridSpacing was set 1.0 Å. The cutoff distance for non-bonded interactions was 10 Å. A smoothing function was employed for the van der Waals interactions at a distance of 8 Å. The pair list of non-bonded interactions was evaluated using a pair distance of 14 Å. The whole receptor was simulated for a total time of 100 ns. WT, R420D and QDA systems were simulated for 100 ns each, applying symmetry restraints.

#### Free energy calculations

From the crystal structure of the whole 5-HT_3_A receptor, a smaller subsystem comprising only TMD and ICD was created. Only the ions needed to ensure the system electroneutrality were introduced. After 2 ns equilibration, we performed a steered MD simulation, slowly dragging one Na^+^ ion (or one Cl^-^) through the pore in order to generate multiple starting configurations suitable for the ABF windows. Each ion was dragged along the TMD pore in about 30 ns. Subsequently, we equilibrated each window for 2 ns prior to free energy calculations. The Adaptive Biasing Force (ABF) method as implemented in NAMD[88–90], was used to calculate the single-ion PMF for Na^+^ and Cl^−^ along a reaction coordinate spanning ~65 Å, sufficient to cover the entire length of the TMD and the upper part of the ICD. The method couples ideas from thermodynamic integration and average force formalisms with unconstrained molecular dynamics and the introduction of an adaptive biasing potential. The average force experienced by the simulated system at any point along a coordinate ξ is estimated from the instantaneous forces experienced by the system at that position. The average forces are accumulated in bins along ξ and are continuously updated as the simulation progresses. The estimated free energy derivative, computed for small intervals of ξ, is canceled by the introduction of an adaptive biasing potential. The application of the adaptive bias allows the system to overcome existing barriers along ξ in the free energy landscape. The simulations were carried out in 1 window of 3 Å length and 11 windows of 5 Å length along the channel axis. The same simulation conditions described above were applied for this calculation. Within each window the average force acting on the selected ion was accumulated in 0.1 Å sized bins, and the biasing force was applied after 800 samples. Positional restraints (with force constant of 3 kcal/mol/Å^2^) were applied to counter-ions so to keep them away from the ion inside the channel and rule out any interference in PMF calculation. A 20 ns MD trajectory was generated for each window, resulting in a total simulation time of 240 ns per ion. Convergence was tested by plotting the PMF profile at different time intervals (S11 Fig).

#### Trajectory analysis

The HOLE[91] program and the MolAxis[92] webserver were used to analyze pore dimension and fenestrations. RMSF and B-factors were calculated using the ptraj[93] software tool. RMSD, ion density and coordination analyses were performed using in-house scripts exploiting the MDAnalysis[94] library. Electrostatic potentials were evaluated through the APBS[95] server. Figures and plots were generated using the UCSF-CHIMERA[96] and MATPLOTLIB[97] software.

## Supporting Information

S1 Fig Root Mean Square Deviation with respect to the starting frame. (TIFF)

S2 Fig Backbone RMSF. The protein average structure, depicted as ribbons, is colored according to backbone RMSF values, from lowest (blue) to highest (red). The thickness of the ribbons is also proportional to RMSF values.
(TIFF)

S3 Fig K108 Side chains conformation. Bottom view of K108 side chain conformations in the (A) 5-HT3A crystal structure and (B) a representative snapshot from the simulation. The protein is depicted as yellow ribbons, while the side chains of K108 and D105 are depicted as sticks.
(TIFF)

S4 Fig Enlarged view of a lateral portal at the ECD-TMD interface. The protein structure is depicted as cartoons, with residue side chains molding the portal walls as sticks. Cys-Loop, β1- β2 loop, C-terminal part of the F-loop, M2-M3 loop and β10-M1 loop are highlighted in blue, magenta, green, yellow and orange, respectively. In background, one channel predicted by MolAxis is depicted as a series of transparent yellow spheres, whose radius is proportional to the accessible space.
(TIFF)

S5 Fig Lateral channels in other members of the Cys-Loop superfamily. Lateral intersubunit pathways from the interior to the exterior of the protein in: (A) 5-HT_3_A, (B) nAchR, (C) GluCl, (D) ELIC, (E) GLIC.
(TIFF)

S6 Fig Average number of ions along the channel axis. Bar histogram of the average number of Na^+^ (blue) and Cl^-^ (red) ions along the channel axis (inside a cylinder of 25 Å radius). Negative values represent the extracellular side of the pore, while positive values represent the intracellular side. Origin is set to the centroid of D271 C_α_ atoms.
(TIFF)

S7 Fig Average ion coordination number along the channel axis. The blue and red curves represent the average number of water and protein side chains atoms, respectively. The values are obtained from the last 5 ns of the ABF simulations.
(TIFF)

S8 Fig RMSD of ICD Portals. Root mean square deviation, with respect to the starting configuration, of the stretch of residues that line the ICD portals (302–308, 409–418) in WT, R420D and QDA systems. In the right subplot is shown the corresponding RMSD distribution.
(TIFF)

S9 Fig Backbone flexibility of ICD portals. Backbone RMSF of the ICD portals in (A) WT, (B) R420D and (C) QDA systems. The protein structure is depicted as cartoons and colored according to the RMSF values.
(TIFF)

S10 Fig Side chain flexibility at ICD portals. View of ICD portals in (A) WT, (B) R420D and (C) QDA systems. Protein is shown as yellow cartoon, and the stretch of residues shaping the portal walls are shown as sticks and colored according to their side chains B-factors. A shift toward red can be appreciated for residue side chains in the post-M3 loop, especially D312.
(TIFF)

S11 Fig Convergence of the potential of mean force. PMFs for Na^+^ and Cl^-^ permeation calculated from sequentially increasing time intervals from the ABF simulations. Changes in the PMF in the last 5ns are below 2 kcal/mol.
(TIFF)

S1 Video Lateral Translocation. (MP4)
