# Supplementary figures and images for: Pathways and Barriers for Ion Translocation through the 5-HT3A Receptor Channel
Source: PLoS One. 2015 Oct 14;10(10):e0140258. doi: 10.1371/journal.pone.0140258 (PMC4605793; doi:10.1371/journal.pone.0140258)

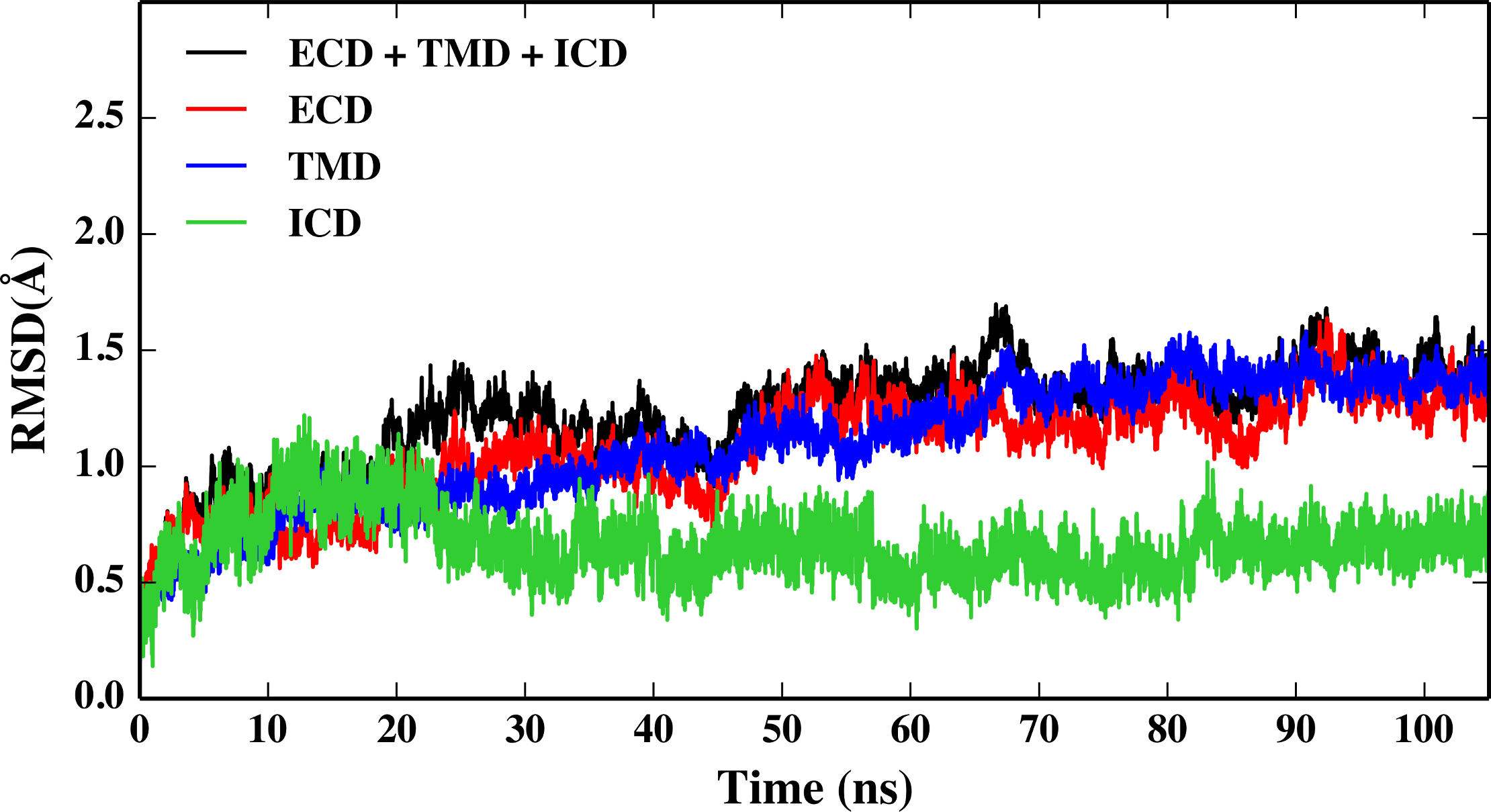

Supplement: S1 Fig — (TIFF) [file pone.0140258.s001.tiff]

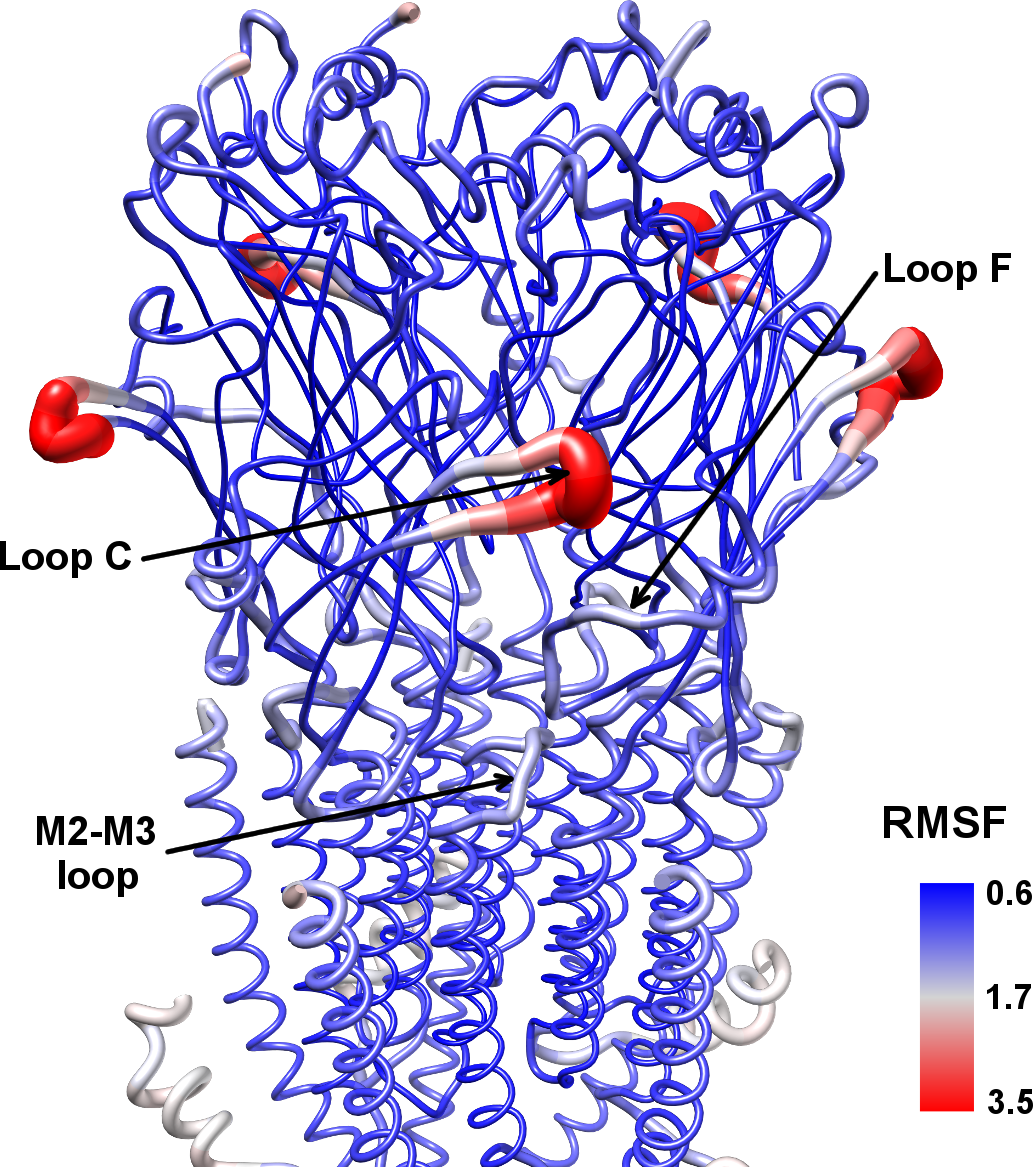

Supplement: S2 Fig — The protein average structure, depicted as ribbons, is colored according to backbone RMSF values, from lowest (blue) to highest (red). The thickness of the ribbons is also proportional to RMSF values. (TIFF) [file pone.0140258.s002.tiff]

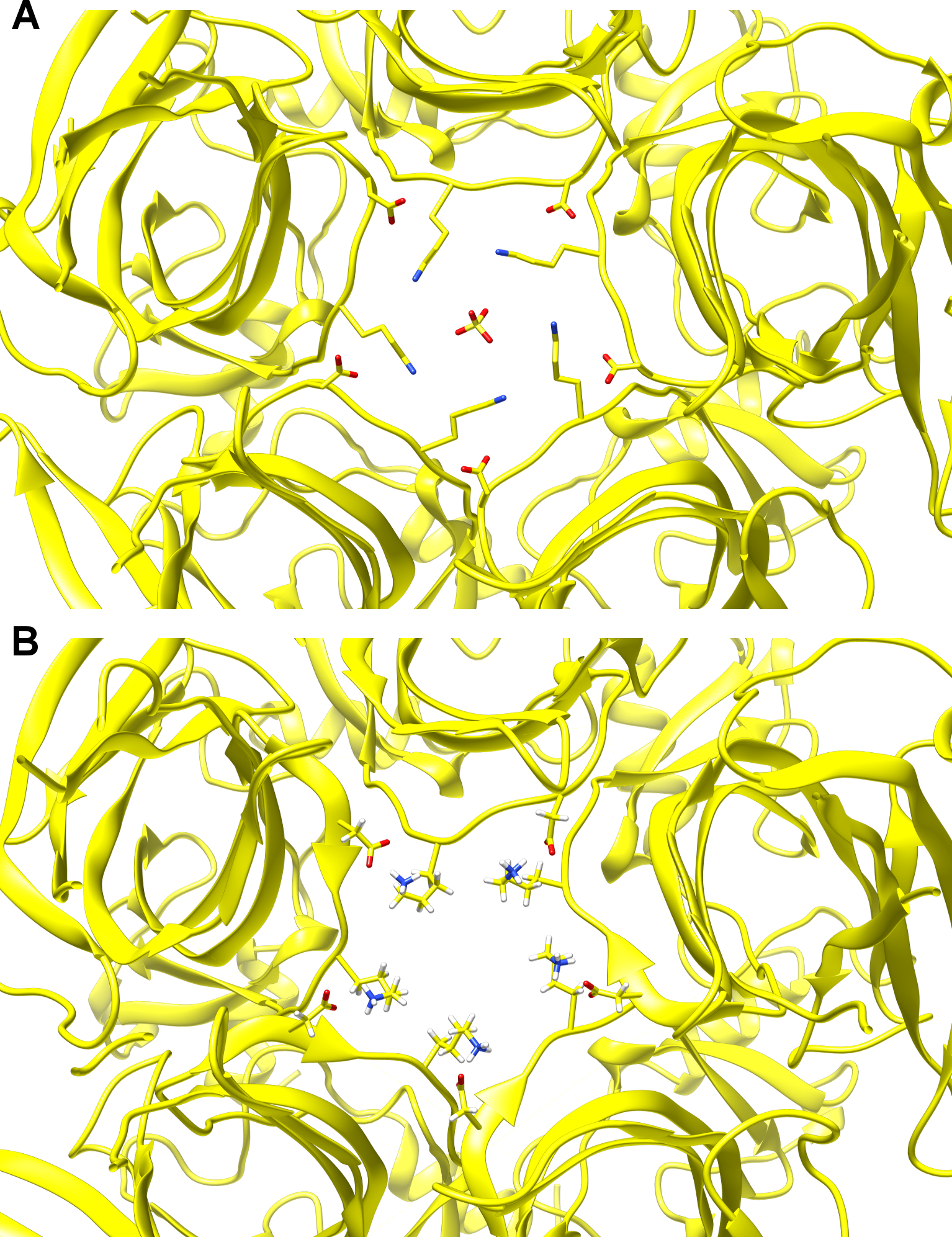

Supplement: S3 Fig — Bottom view of K108 side chain conformations in the (A) 5-HT3A crystal structure and (B) a representative snapshot from the simulation. The protein is depicted as yellow ribbons, while the side chains of K108 and D105 are depicted as sticks. (TIFF) [file pone.0140258.s003.tiff]

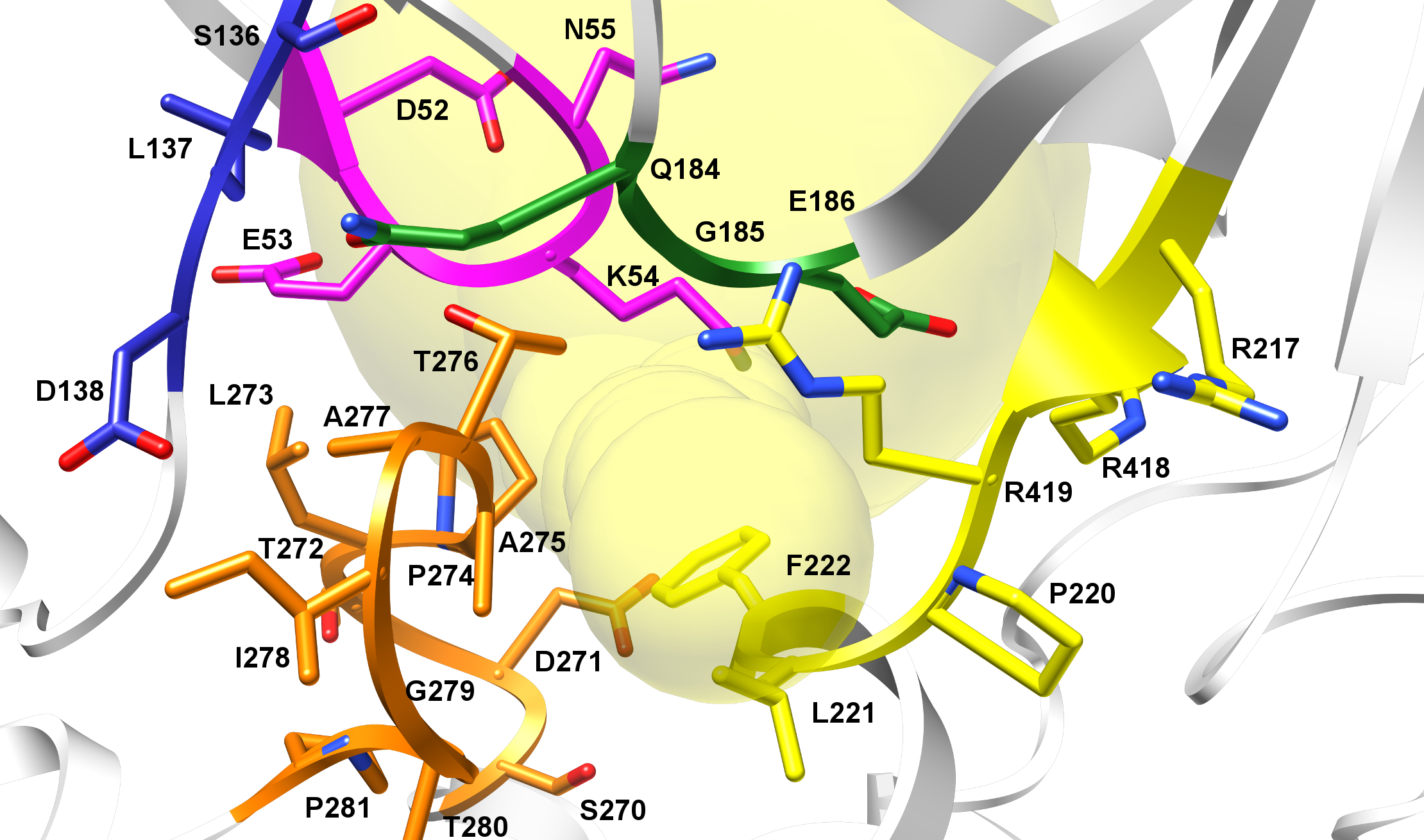

Supplement: S4 Fig — The protein structure is depicted as cartoons, with residue side chains molding the portal walls as sticks. Cys-Loop, β1- β2 loop, C-terminal part of the F-loop, M2-M3 loop and β10-M1 loop are highlighted in blue, magenta, green, yellow and orange, respectively. In background, one channel predicted by MolAxis is depicted as a series of transparent yellow spheres, whose radius is proportional to the accessible space. (TIFF) [file pone.0140258.s004.tiff]

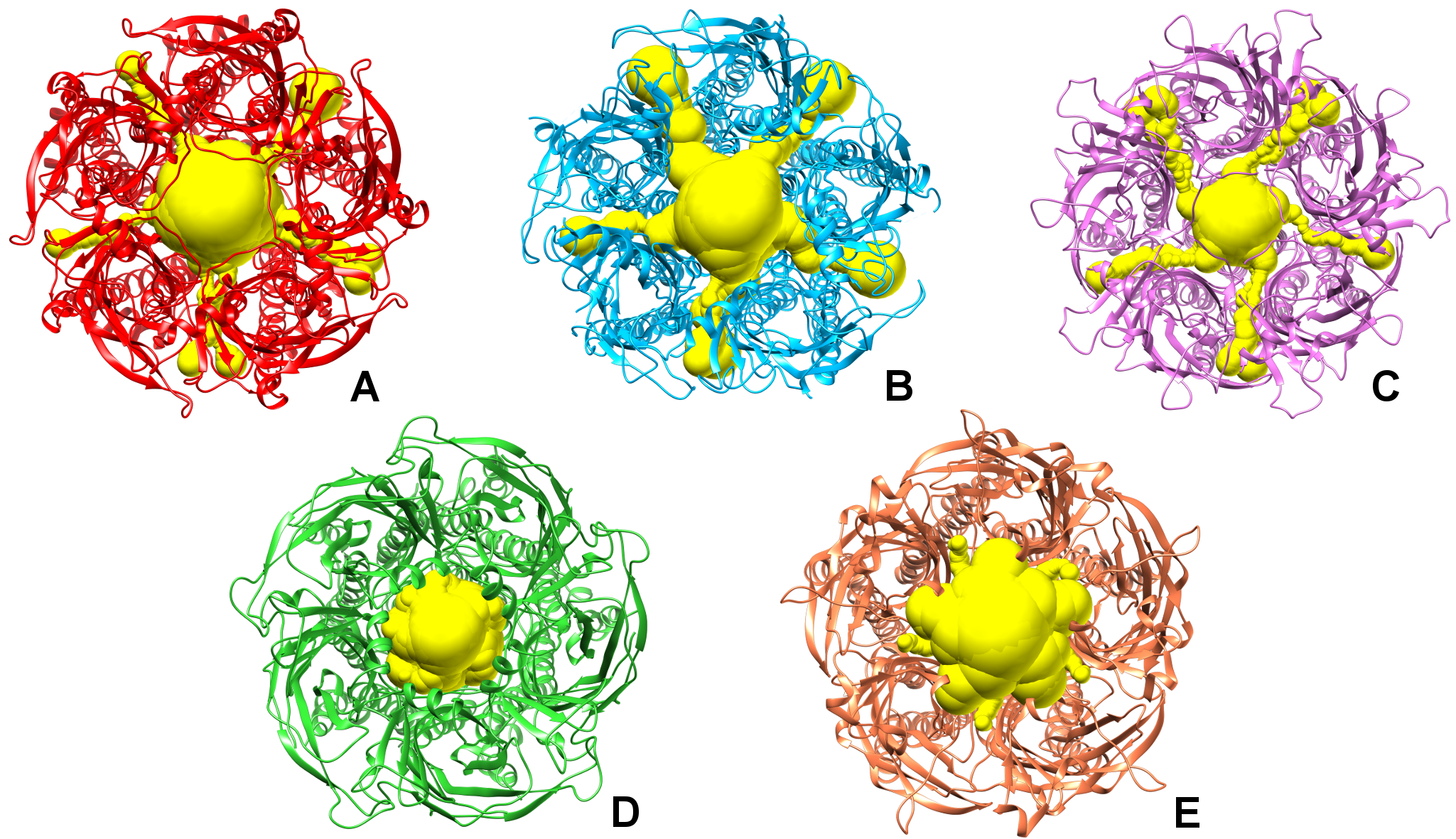

Supplement: S5 Fig — Lateral intersubunit pathways from the interior to the exterior of the protein in: (A) 5-HT3A, (B) nAchR, (C) GluCl, (D) ELIC, (E) GLIC. (TIFF) [file pone.0140258.s005.tiff]

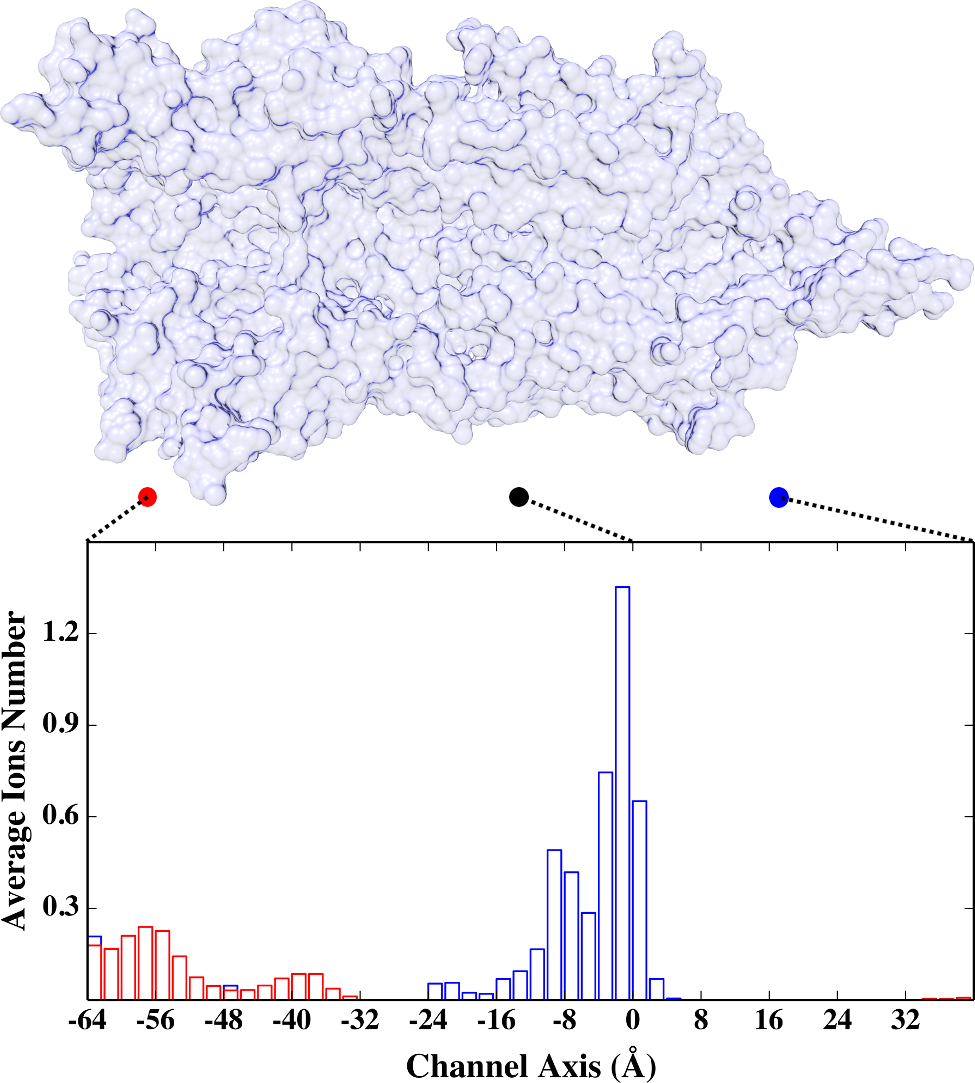

Supplement: S6 Fig — Bar histogram of the average number of Na+ (blue) and Cl- (red) ions along the channel axis (inside a cylinder of 25 Å radius). Negative values represent the extracellular side of the pore, while positive values represent the intracellular side. Origin is set to the centroid of D271 Cα atoms. (TIFF) [file pone.0140258.s006.tiff]

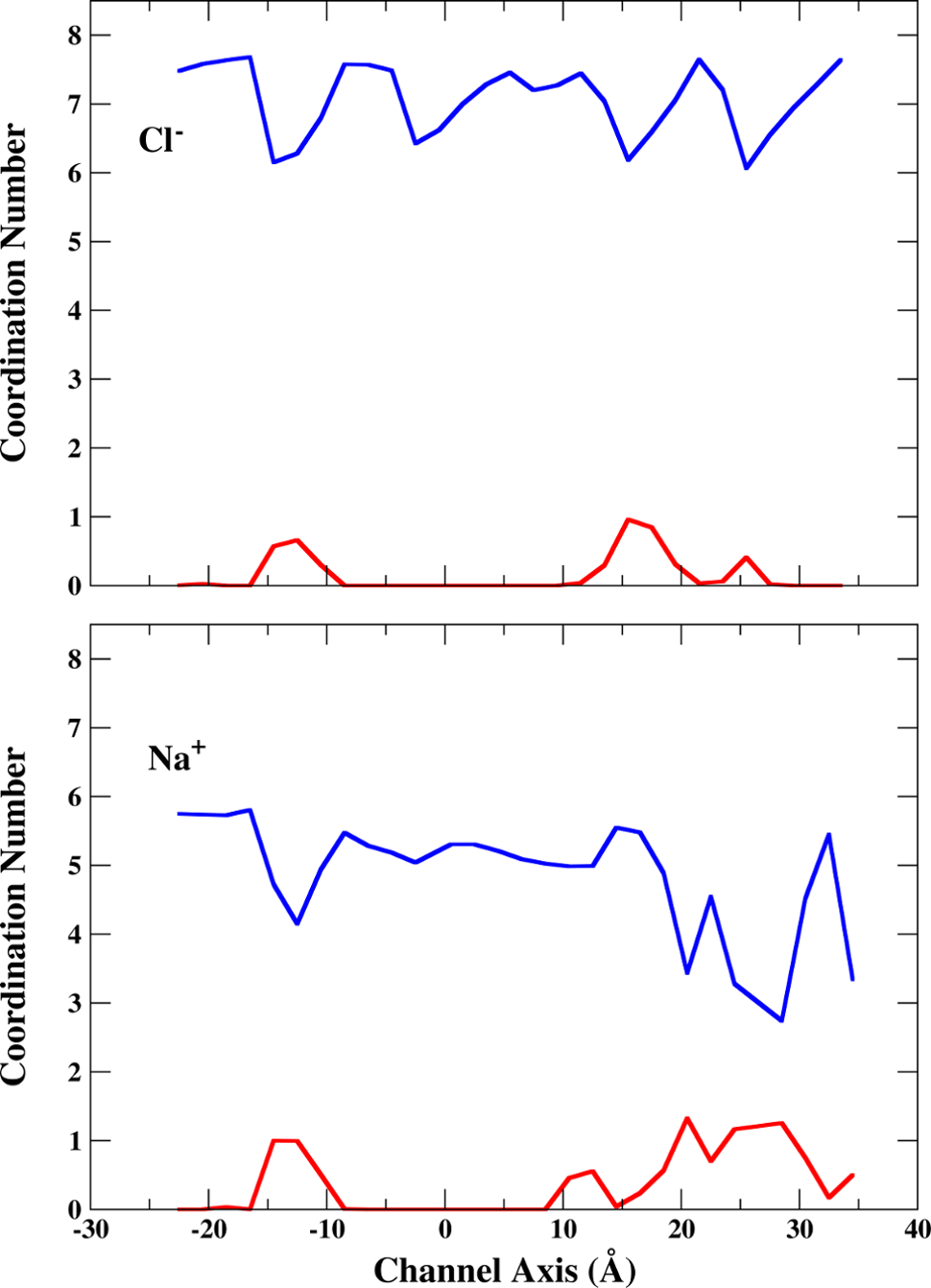

Supplement: S7 Fig — The blue and red curves represent the average number of water and protein side chains atoms, respectively. The values are obtained from the last 5 ns of the ABF simulations. (TIFF) [file pone.0140258.s007.tiff]

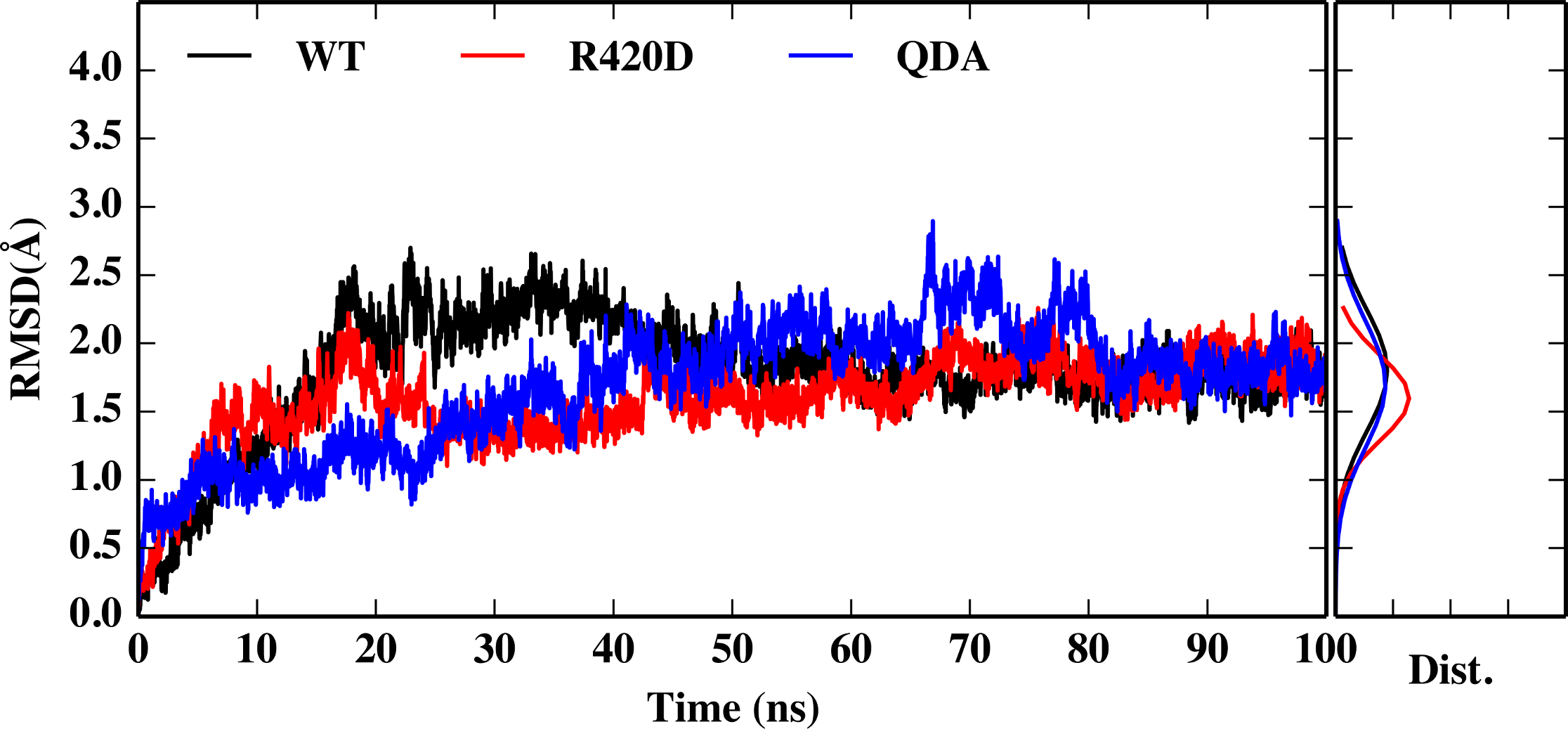

Supplement: S8 Fig — Root mean square deviation, with respect to the starting configuration, of the stretch of residues that line the ICD portals (302–308, 409–418) in WT, R420D and QDA systems. In the right subplot is shown the corresponding RMSD distribution. (TIFF) [file pone.0140258.s008.tiff]

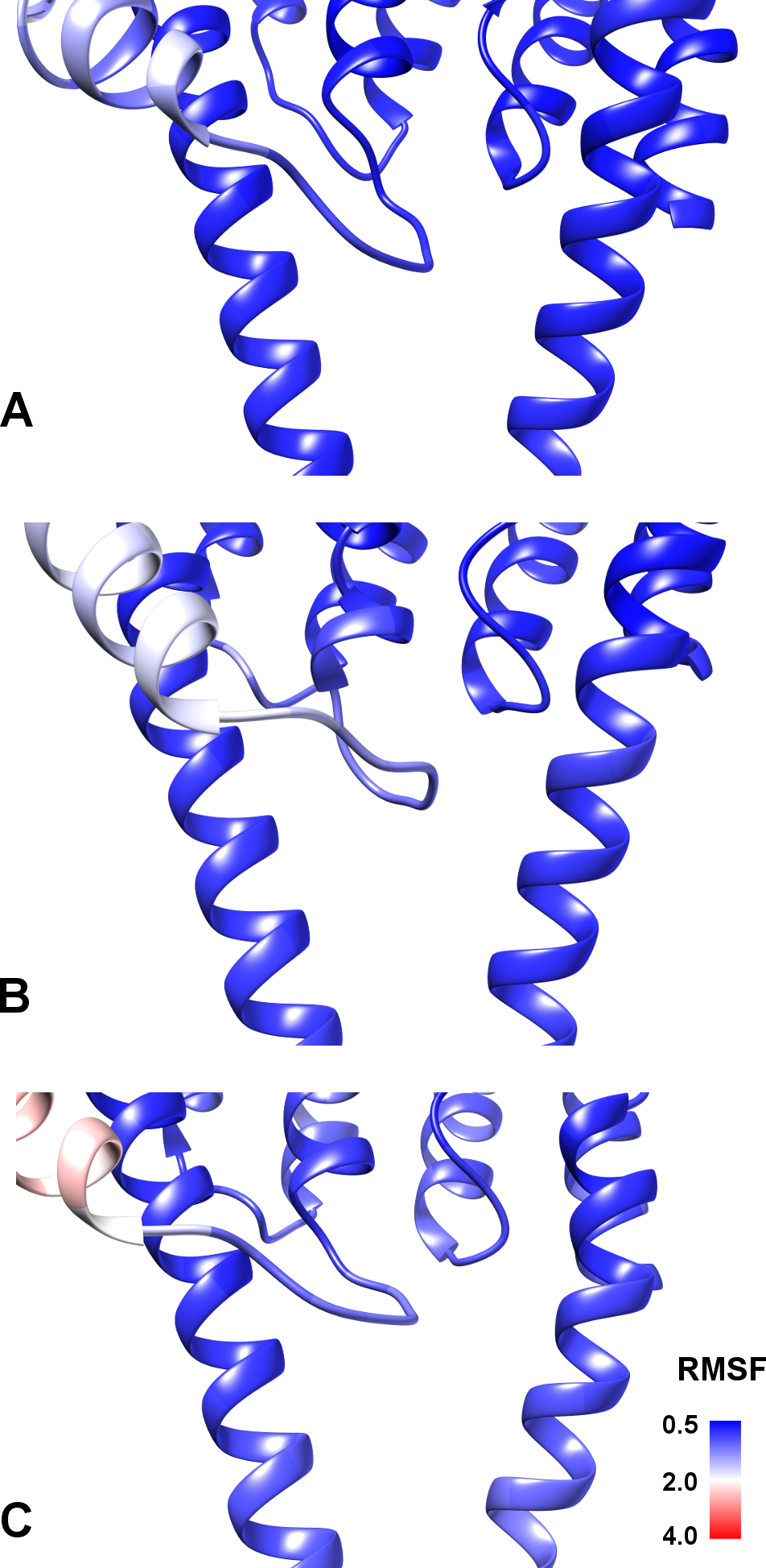

Supplement: S9 Fig — Backbone RMSF of the ICD portals in (A) WT, (B) R420D and (C) QDA systems. The protein structure is depicted as cartoons and colored according to the RMSF values. (TIFF) [file pone.0140258.s009.tiff]

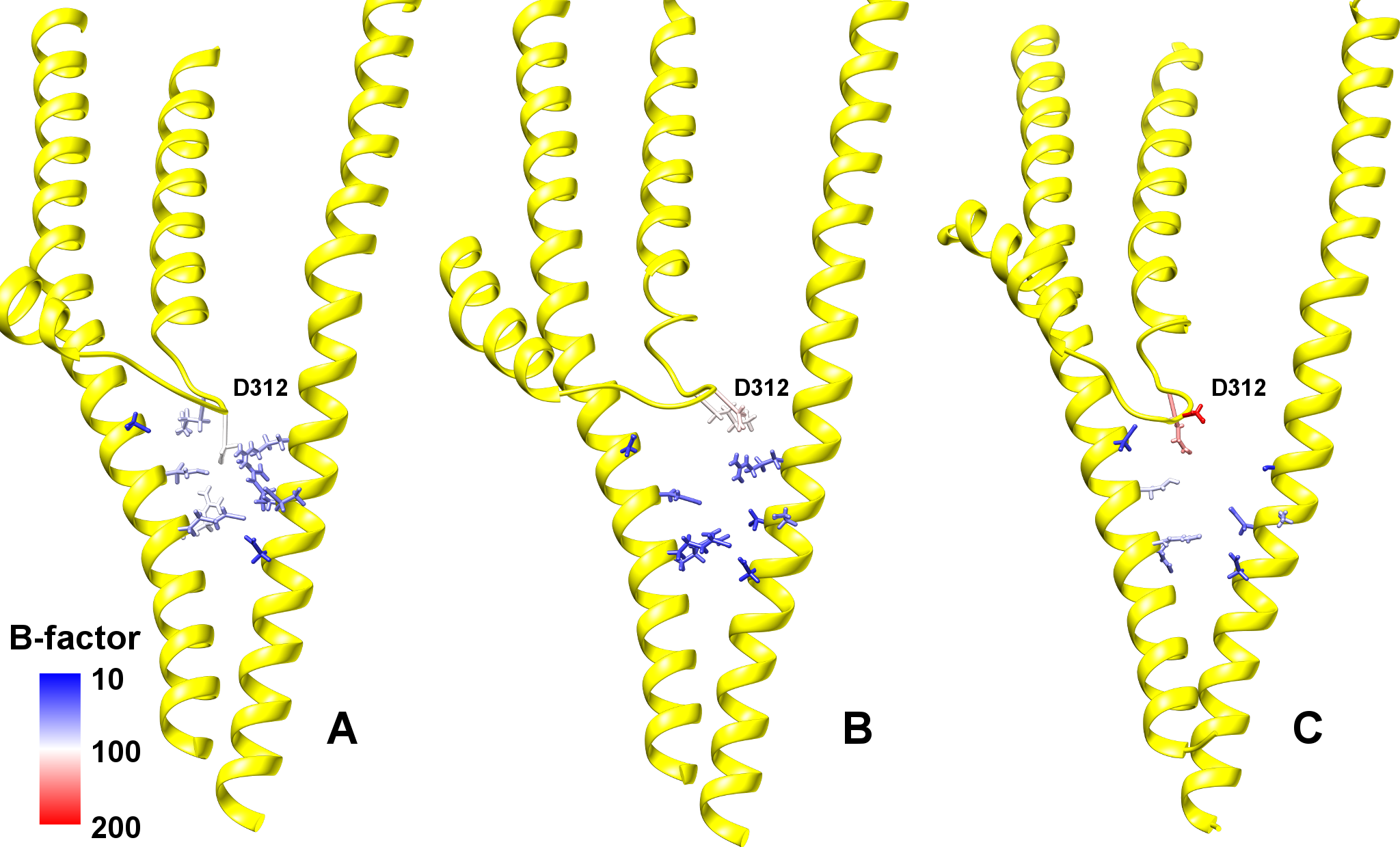

Supplement: S10 Fig — View of ICD portals in (A) WT, (B) R420D and (C) QDA systems. Protein is shown as yellow cartoon, and the stretch of residues shaping the portal walls are shown as sticks and colored according to their side chains B-factors. A shift toward red can be appreciated for residue side chains in the post-M3 loop, especially D312. (TIFF) [file pone.0140258.s010.tiff]

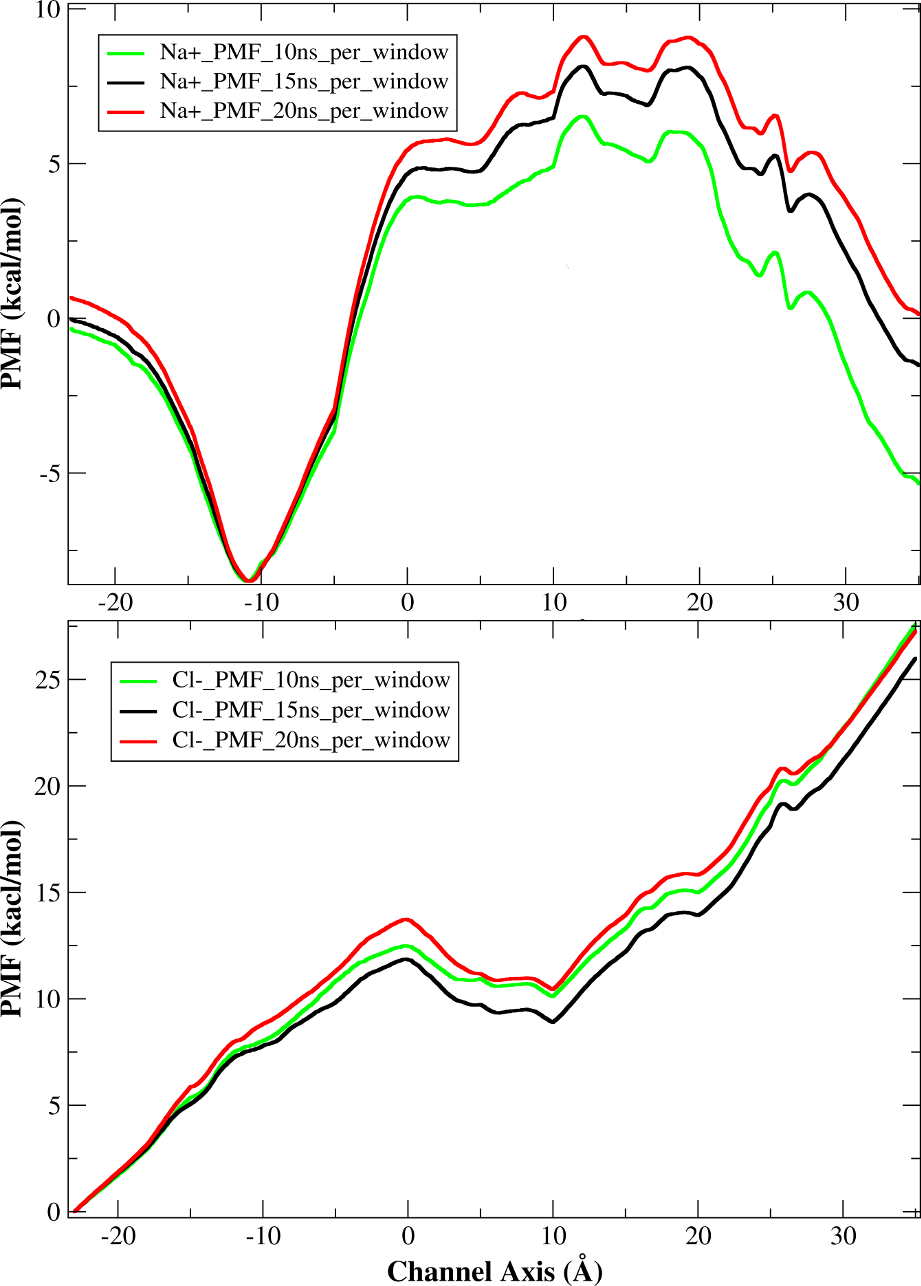

Supplement: S11 Fig — PMFs for Na+ and Cl- permeation calculated from sequentially increasing time intervals from the ABF simulations. Changes in the PMF in the last 5ns are below 2 kcal/mol. (TIFF) [file pone.0140258.s011.tiff]
